# The transcriptome analysis of the *Arabidopsis thaliana* in response to the *Vibrio vulnificus* by RNA-sequencing

**DOI:** 10.1371/journal.pone.0225976

**Published:** 2019-12-16

**Authors:** Yong-Soon Park, Seon-Kyu Kim, Seon-Young Kim, Kyung Mo Kim, Choong-Min Ryu

**Affiliations:** 1 Biotechnology Research Institute, College of Natural Sciences, Chungbuk National University, Cheongju, South Korea; 2 Molecular Phytobacteriology Laboratory, Infection Disease Research Center, KRIBB, Daejeon, South Korea; 3 Personalized Genomic Medicine Research Center, KRIBB, Daejeon, South Korea; 4 Genome Editing Research Center, KRIBB, Daejeon, South Korea; 5 Department of Functional Genomics, University of Science and Technology (UST), Daejeon, South Korea; 6 Microbial Resource Center, KRIBB, Jeongeup, South Korea; 7 Biosystem and Bioengineering Program, University of Science and Technology (UST), Daejeon, South Korea; Fujian Agriculture and Forestry University, CHINA

## Abstract

Because of the recent increase in the demand for fresh produce, contamination of raw food products has become an issue. Foodborne diseases are frequently caused by the infection of leguminous plants by human bacterial pathogens. Moreover, contamination by *Vibrio cholerae*, closely related with *Vibrio vulnificus*, has been reported in plants and vegetables. Here, we investigated the possibility of *Vibrio vulnificus* 96-11-17M, an opportunistic human pathogen, to infect and colonize *Arabidopsis thaliana* plants, resulting in typical disease symptoms at 5 and 7 days post-inoculation *in vitro* and *in planta* under artificial and favorable conditions, respectively. RNA-Seq analysis revealed 5,360, 4,204, 4,916 and 3,741 differentially expressed genes (DEGs) at 12, 24, 48 and 72 h post-inoculation, respectively, compared with the 0 h time point. Gene Ontology analysis revealed that these DEGs act in pathways responsive to chemical and hormone stimuli and plant defense. The expression of genes involved in salicylic acid (SA)-, jasmonic acid (JA)- and ethylene (ET)-dependent pathways was altered following *V*. *vulnificus* inoculation. Genetic analyses of *Arabidopsis* mutant lines verified that common pathogen-associated molecular pattern (PAMP) receptors perceive the *V*. *vulnificus* infection, thus activating JA and ET signaling pathways. Our data indicate that the human bacterial pathogen *V*. *vulnificus* 96-11-17M modulates defense-related genes and host defense machinery in *Arabidopsis thaliana* under favorable conditions.

## Introduction

Over the past few decades, the availability of fresh foods for optimum human health has received considerable attention. Although suppliers have put tremendous effort into reducing the contamination of fresh foods, the number of cases related to contaminated food products has been increasing [1,2]. Contamination of raw food products is mainly caused by bacteria, viruses and parasites. Bacteria are prokaryotic microorganisms that inhabit and adapt to soil, water and acidic hot springs and function as symbionts or pathogens in animals and plants [1]. Human bacterial pathogens including *Salmonella* [3] and *Escherichia coli* O157 contaminate fresh fruits and vegetables [4]. Between 2000 and 2008, a number of cases of foodborne diseases caused by *Salmonella* spp. and norovirus were reported in the US, and *Salmonella* has been ranked as the major bacterial pathogen responsible for the hospitalization and death of humans [5]. In addition to the direct impact on human health, these pathogens also cause substantial economic losses, as contaminated produce has to be recalled by the supplier. For example, the recall of contaminated spinach resulted in the loss of $350 million in the USA in 2006 [6].

Plants may be contaminated at any stage of growth or during post-harvest processing. Human pathogen population increases both before the harvest of plants [7,8] as well as post-harvest [7]. Sterilization and sanitation are used to eliminate the contamination of fresh produce by decreasing the population of human pathogens in plants. However, despite extensive efforts, internalized human pathogens cannot be completely eliminated from plants [9,10]. Thus, understanding the interaction between human pathogens and plants is critical for controlling the outbreak of foodborne illnesses.

To date, *V*. *vulnificus* is a Gram-negative halophilic bacterium and a lactose-positive opportunistic human pathogen [11]. *V*. *vulnificus* grows in aquatic environments worldwide, especially in warm waters in tropical and subtropical regions [12,13]. *V*. *vulnificus* has been classified into three distinct biotypes, based on its lipopolysaccharide (LPS) antigens, expression of capsule and host range [14,15]. Biotype 1 has been predominantly associated with shellfish colonization and human diseases and was initially divided into five LPS groups [16]. Biotype 2 mostly infects marine vertebrates (e.g., eels) and possesses a single type of LPS antigen known as serogroup E [17]. Biotype 3 has been relatively recently updated in Israel fish farms by wound infection with live fish [15]. Infection by *V*. *vulnificus* occurs via two main routes: 1) septicaemia resulting from the consumption of *V*. *vulnificus*-contaminated raw seafood; and 2) wound infection [18]. Recently, *V*. *vulnificus* infection has been reported to cause gastroenteritis [18].

It is known that *V*. *cholerae* shares many similarities with *V*. *vulnificus* [19]. Moreover, contamination of *V*. *cholerae* has been reported in diverse plants and vegetables for a long time [20,21,22,23]. However, despite its ability to cause severe disease, *V*. *vulnificus* has not yet been reported to cause any plant disease. This notion prompts us to test the possibility of *V*. *vulnificus* causes disease in plants and how it modulates the plant defense responses under artificial and favorable conditions. To investigate further experiments, we first identified the strains of *V*. *vulnificus* that clearly contribute to the development of pathogenicity in *Arabidopsis in vitro*. Among the tested strains, *V*. *vulnificus* 96-11-17M was selected for further analyses. Next, we examined the disease severity and pathogen population density in *Arabidopsis* plants inoculated with this strain *in planta*. To obtain molecular evidence, we performed RNA-sequencing (RNA-Seq) analysis of *Arabidopsis* plants infiltrated with *V*. *vulnificus* 96-11-17M *in planta*. The results showed that *V*. *vulnificus* 96-11-17M infection altered the transcript levels of genes involved in salicylic acid (SA)-, jasmonic acid (JA)- and ethylene (ET)-dependent pathways at the tested time points relative to time zero (control). Furthermore, several differentially expressed genes (DEGs) identified in *Arabidopsis* plants inoculated with *V*. *vulnificus* 96-11-17M significantly overlapped with those identified in *Arabidopsis* plants inoculated with *Pseudomonas syringae* pv. *maculicola* ES4326, a common plant bacterial pathogen. Taken together, our results provide that *V*. *vulnificus* modulates defense-related genes and host defense machinery in *Arabidopsis thaliana* under favorable conditions used in this study.

## Materials and methods

### Plant materials

#### In vitro

Seeds of *Arabidopsis* ecotype Col-0 were surface-sterilized with 6% sodium hypochlorite for 5 min and then washed five times with sterile distilled water. The sterilized seeds were plated on half-strength Murashige and Skoog (0.5× MS) medium supplemented with 3% sucrose and 0.6% plant agar. Seeds were germinated and incubated in a growth chamber at 24 ± 2°C under a 12 h light/12 h dark photoperiod and fluorescent light (7,000 lux light intensity) for 3 days. The germinated seeds were transferred to fresh 0.5× MS plates and incubated for 2 weeks under the same conditions for further experiments.

#### In planta

Seeds of wild-type (Col-0) and mutant (*fls2*, *efr1*, *fls2/efr1*, NahG, *sid2*, *npr1*, *jar1* and *etr1*) plants were planted in soil-less potting medium (Punong, Gyeongju, South Korea). Plants were grown at 24 ± 2°C under a 8 h light/16 h dark photoperiod and fluorescent light (7,000 lux light intensity) for 3 weeks.

### Disease severity and population *in vitro*

To perform *in vitro* assays, 2-week-old *Arabidopsis* seedlings were separately drop-inoculated with bacterial suspensions of four strains of *V*. *vulnificus* including 96-11-17M, A1402, CMCP6 and YJ016 at an optical density at 600 nm (OD_600_) of 2.0 and with *Pseudomonas syringae* pv. tomato DC3000 (positive control; OD_600_ = 1). Briefly, bacterial strains were picked from each glycerol stock at -70°C, streaked on LB agar medium containing 2% NaCl (Sigma, CA, USA) and 5 μg/ml rifampicin (Yuhan Corporation, Seoul, South Korea) and incubated at 30°C for 2 days. *P*. *syringae* pv. *tomato* DC3000 was also steaked on LB agar medium containing 5 μg/ml rifampicin and incubated at 30°C for 2 days. After incubation, colonies were scripted by plastic bar using sterilized 10% PBS (Bioneer, Daejeon, South Korea). The 10% PBS buffer contained NaCl (13.7 mM), KCl (0.27 mM), Na_2_HPO_4_ (1 mM) and KH_2_PO_4_ (0.18 mM) at pH 7.4. The inoculated plants were incubated in a growth chamber for 5 days and then scored for disease severity. Plants inoculated with *P*. *syringae* pv. *tomato* DC3000 re-suspended in 10% PBS were scored on a scale from 0 to 5, where 0 indicated no symptoms, 1 indicated the development of yellowish color, 2 indicated chlorosis only, 3 indicated necrosis and chlorosis, 4 indicated partial necrosis of the inoculated area and 5 indicated complete necrosis of the inoculated area [24]. The 10% PBS used a negative control because all strains of bacteria were resuspened in 10% PBS.

For population quantification *in vitro* assays, leaves at the same stage and age were collected at 5 days after drop-inoculation of *V*. *vulnificus* 96-11-17M and *P*. *syringae* pv. *tomato* DC3000, homogenized, spread on LB agar medium containing 2% NaCl and 5 μg/ml rifampicin and LB agar medium containing 5 μg/ml rifampicin, respectively, and finally incubated at 30°C for 2 days. *P*. *syringae* pv. *tomato* DC3000 was also steaked on and incubated at 30°C for 2 days. The number of *V*. *vulnificus* 96-11-17M and *P*. *syringae* pv. *tomato* DC3000 colonies was counted at 2 days after incubation.

### Disease symptom and population of *V*. *vulnificus* 96-11-17M *in planta*

**Fig 1:**
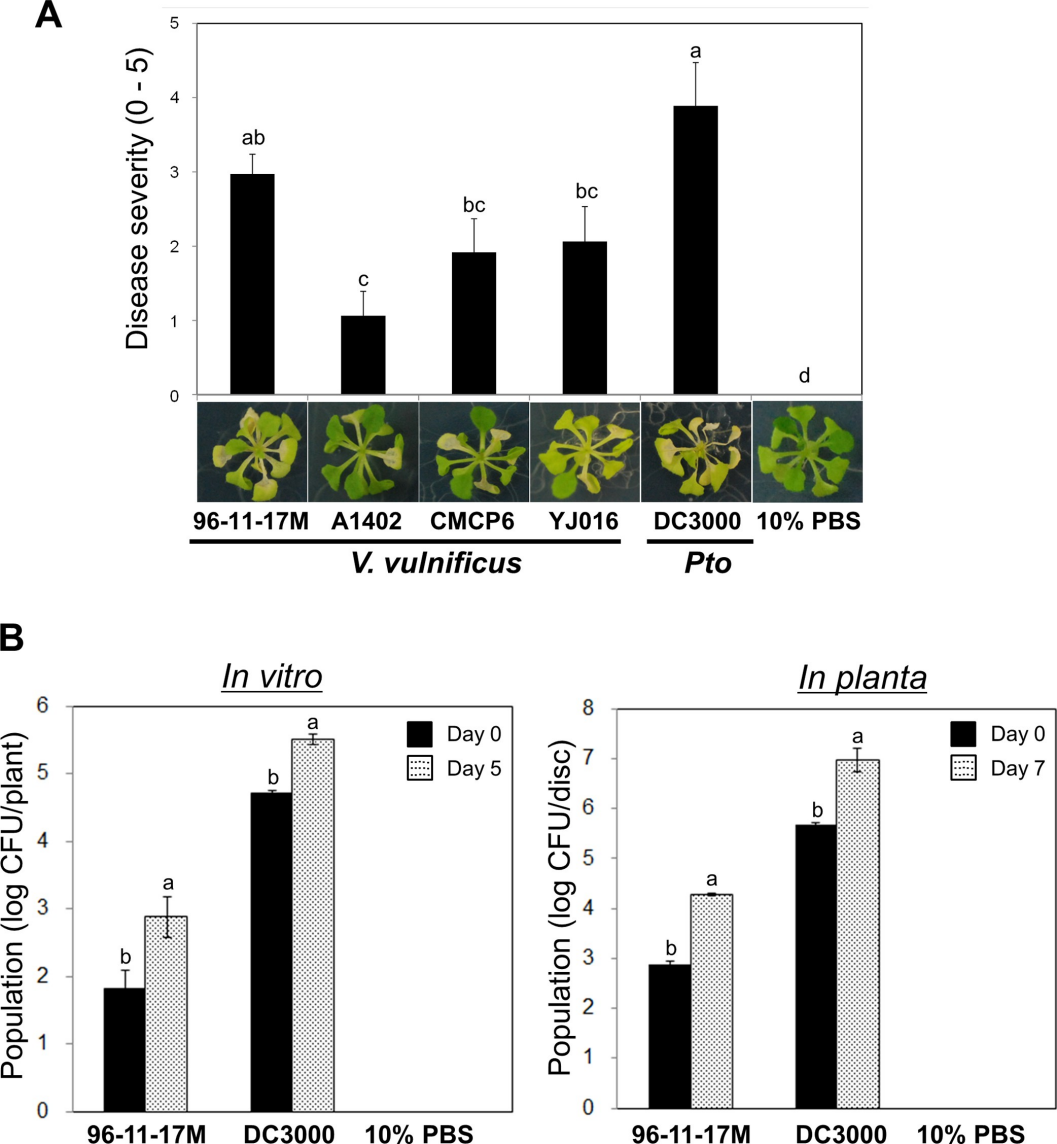
Pathogenicity of *Vibrio vulnificus* in *Arabidopsis thaliana*. (A) Assessment of disease severity in *Arabidopsis* plants upon drop-inoculation with four *V*. *vulnificus* strains, *Pseudomonas syringae* pv. *tomato* (*Pto*) DC3000 (positive control) or 10% PBS (negative control). Disease symptoms were clearly observed in *V*. *vulnificus* -inoculated plants. (B) Total population density of *V*. *vulnificus* 96-11-17M. Left panel: initial and final population density measured at 5 days post-inoculation *in vitro*. Right panel: *V*. *vulnificus* 96-11-17M population monitored for 7 days post-infiltration *in planta*.

The suspensions of *V*. *vulnificus* 96-11-17M was prepared as described in above part. To determine the population density of *V*. *vulnificus* 96-11-17M *in planta*, leaves of 3-week-old wild-type (Col-0) seedlings only (Fig 1B) or Col-0, *fls2*, *efr1* and *fls2/efr1* (Fig 3C) or Col-0, NahG, *sid2*, *npr1*, *jar1* and *etr1* mutant seedlings (Fig 3D), were infiltrated with *V*. *vulnificus* 96-11-17M (OD_600_ = 1) and collected at 3, 4 or 7 days post-infiltration. The collected leaves were homogenized and resuspended in 10% PBS. Subsequently, the resuspended samples were spread on LB agar medium containing 2% NaCl and 5 μg/ml rifampicin and incubated at 30°C. The number of *V*. *vulnificus* 96-11-17M colonies was counted at 2 days after incubation. Disease symptom was observed by *V*. *vulnificus* 96-11-17M infiltration *in planta* and photograph was taken at 7 days after infiltration.

### Total RNA extraction, cDNA synthesis and qRT-PCR

Leaves of 3-week-old wild-type plants infiltrated with *V*. *vulnificus* 96-11-17M (OD_600_ = 1 in 10% PBS) were harvested at 0, 12, 24, 48 and 72 h post-infiltration and immediately frozen in liquid nitrogen. Subsequently, the leaf tissues were ground to a fine powder using a mortar and pestle. Total RNA was isolated from 100 mg ground tissue using RNeasy^®^ Plus Mini kit, according to the manufacturer’s protocol (Qiagen, CA, USA). First-strand cDNA was synthesized using 2 μg total RNA, oligo-dT primer, dNTPs and Moloney murine leukaemia virus reverse transcriptase (M-MLV RT; Enzynomics, Daejeon, South Korea). To validate the RNA-Seq data, qRT-PCR was performed using a Chromo4 Real-time PCR System (Bio-RAD, CA, USA). Each reaction mixture contained 2× Brilliant SYBR Green Supermix (Bio-RAD, CA, USA), cDNA template and 0.5 μM gene-specific primers. Sequences were amplified using the following conditions: initial denaturation at 95°C for 10 min, followed by 44 cycles of denaturation at 95°C for 30 sec, annealing at 60°C for 30 sec and extension at 72°C for 42 sec. Gene expression levels were normalized relative to the expression of *AtACT2* (GenBank accession no. Q96292). Primer sets used in this study are listed in S1 Table.

### Construction of RNA-Seq library

Leaves of 3-week-old wild-type *Arabidopsis* seedlings were infiltrated with *V*. *vulnificus* 96-11-17M (OD_600_ = 1 in 10% PBS) and collected at 0, 12, 24, 48 and 72 h post-infiltration; two independent replicates were performed *in planta* for this experiment. Intact total RNA was extracted from the infiltrated leaves, as described above. The quality and integrity of RNA were confirmed by agarose gel electrophoresis, and unwanted contamination with proteins and solvents was assessed using a Nanodrop spectrophotometer (Nanodrop Technologies, Inc., DE, USA). An RNA-Seq library was constructed using TruSeq RNA Sample Preparation kit v2 (Illumina, CA, USA), according to the manufacturer’s guidelines. Briefly, mRNA was purified from total RNA using magnetic beads attached to specific poly oligo-dT primers. The purified mRNA was then fragmented and used as template for cDNA synthesis. The cDNA was ligated to adapters, and the fragments were amplified by PCR. The RNA-Seq library was sequenced to generate paired-end (2 × 100 bp) reads using the Illumina Hiseq-2000 system (Illumina, CA, USA) at the Human Derived Material Center of KRIBB, Daejeon, South Korea.

### RNA-Seq data processing and statistical analysis

To analyze the RNA-Seq data, the reference genome sequence of *A*. *thaliana w*as first obtained from The Arabidopsis Information Resource (TAIR, version 10, assembly ID: TAIR10). The bowtie2-build component of Bowtie2 (ver. 2.0) and SAMtools (ver. 0.1.18) were used for reference genome indexing. Reads from *V*. *vulnificus*-infiltrated samples at different time points were mapped onto the reference genome (ver. 2.0) using the Tophat2 reference genome mapper. The RNA-Seq data generated in this study were deposited at the NCBI Gene Expression Omnibus (GEO) public database under the accession number GSE61418 and data were publicly available.

To analyze the transcriptome, a hierarchical clustering algorithm that uses a centered correlation coefficient as the measure of similarity and complete linkage clustering was applied, as described previously [25]. When performing cluster analysis, the value of fragments per kilobase of exon per million reads (FPKM) was calculated for each sample to estimate gene expression levels. The FPKM data were normalized using quantile normalization in R (version 3.0.1). The FPKM values were log2-transformed and median-centered across genes and samples. To assess differences in gene expression patterns between samples, scatterplot matrices were produced using Pearson correlation tests. Gene set enrichment analysis was carried out to identify the most significant gene sets associated with biological processes. The significance of over-represented gene sets was estimated using Fisher’s exact test. Gene set enrichment analysis was performed using DAVID Bioinformatics Resources (ver. 6.7).

### Statistical analysis

Analysis of variance (ANOVA) of experimental data sets was performed using JMP software version 5.0 (SAS Institute, Cary, NC, USA). Significant effects of treatment were determined based on the magnitude of the F-value (*P* = 0.05). When a significant F-test was obtained, separation of means was accomplished using Fisher’s protected least significant difference test at *P* = 0.05.

## Results

### *V*. *vulnificus* causes disease in *Arabidopsis*

To assess whether the human pathogenic marine bacterium *V*. *vulnificus* causes disease in *A*. *thaliana*, we drop-inoculated *Arabidopsis* leaves with suspensions of four *V*. *vulnificus* strains *in vitro*. All of the tested strains produced typical disease symptoms in *Arabidopsis* leaves similar to those caused by *P*. *syringae* pv. *tomato* (*Pto*) DC3000 (positive control), while no disease symptoms were observed in *Arabidopsis* leaves inoculated with 10% phosphate buffer saline (PBS) (negative control) (Fig 1A). After selection of candidate strain for further experiments, we inoculated *V*. *vulnificus* 96-11-17M onto *A*. *thaliana* leaves, and incubated 5 days in the chamber. Inoculated leaves were collected, homogenized and spread on LB agar medium supplemented with 2% NaCl and 5 ㎍/ml rifampicin. The plates were incubated at 30°C for 2 days. A colony selected from the plates after incubation was used for preparing bacterial suspension (OD_600_ = 2.0). We drop-inoculated with this bacterial suspension onto 2-week-old-*Arabidopsis* leaves and confirmed the same disease symptom at 5 days after inoculation. To confirm that *V*. *vulnificus* colonized *Arabidopsis* plants, we monitored the total bacterial population density after drop-inoculation with *V*. *vulnificus* 96-11-17M and *Pto* DC3000 in the same age and stage leaves. The population density of *V*. *vulnificus* 96-11-17M and *Pto* DC3000 was approximately 10-fold higher on day 5 than on day 0, whereas the negative control showed no *V*. *vulnificus* colonies (Fig 1B, *in vitro*). For consistent results, we infiltrated with *V*. *vulnificus* 96-11-17M and *Pto* DC3000 in *Arabidopsis* plants grown in soil; this was accompanied by a 15-fold increase in the total population density of *V*. *vulnificus* 96-11-17M and *Pto* DC3000 (Fig 1B, *in planta*). These results indicate that *V*. *vulnificus* causes disease in *A*. *thaliana*.

### *V*. *vulnificus* modulates the expression levels of *Arabidopsis* genes

**Fig 2:**
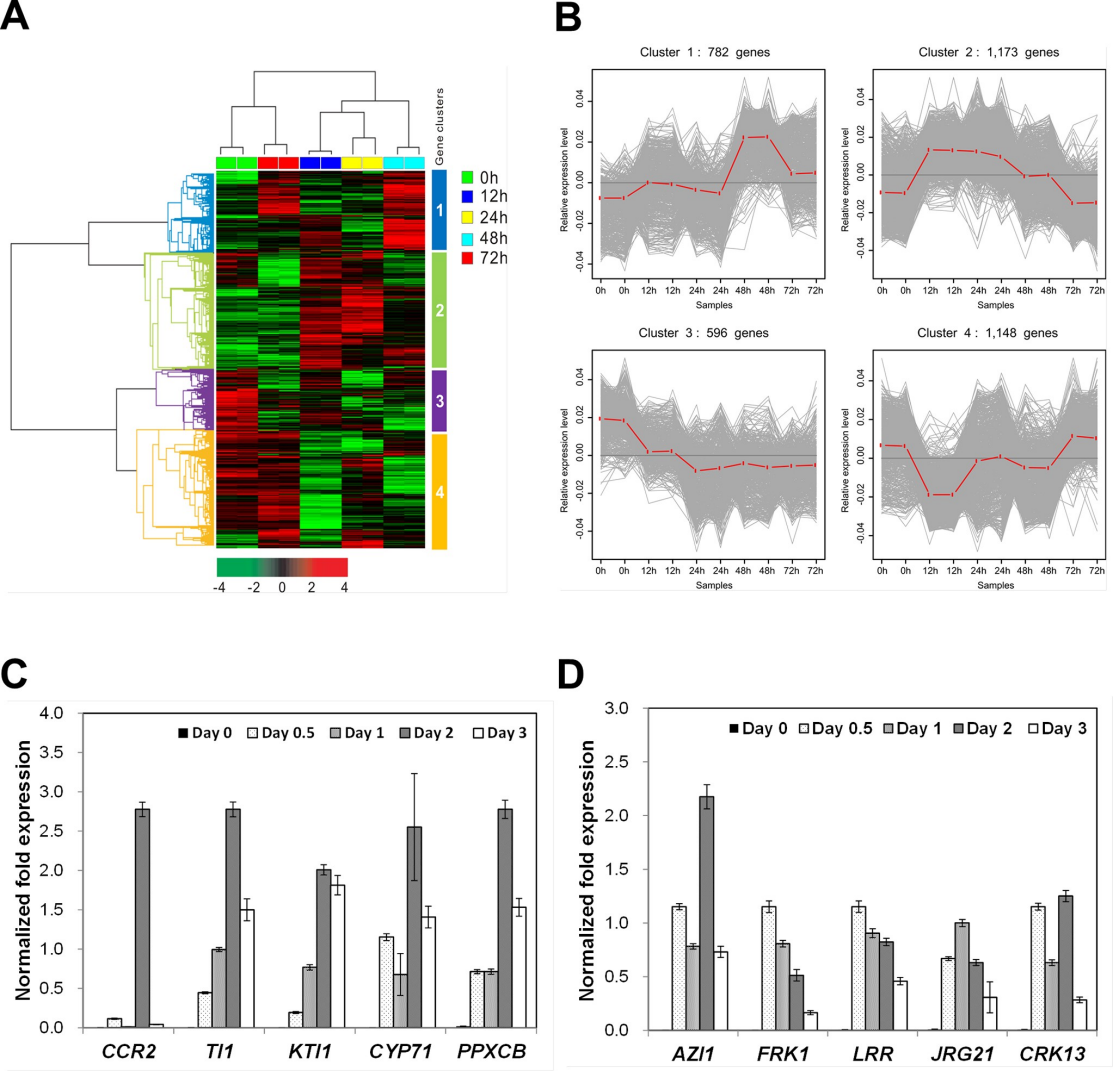
Alteration of transcripts in *A*. *thaliana* in response to *V*. *vulnificus* 96-11-17M infection. (A) Heat map showing the expression of *Arabidopsis* genes at various time points following pathogen inoculation. Gene expression patterns were obtained by performing unsupervised hierarchical cluster analysis, which divided genes into four independent clusters. A total of 3,711 genes with expression values that had a standard deviation of at least 0.7 were selected. Red and green colors indicate high and low expression levels, respectively. (B) Relative expression patterns of *Arabidopsis* genes after *V*. *vulnificus* 96-11-17M inoculation. Grey lines indicate the expression intensities of each gene, and red lines indicate the mean expression levels of all genes at the same time point. (C) Validation of the expression levels of five selected cluster 1 genes by quantitative real-time PCR (qRT-PCR). *CCR*: cold, circadian rhythm and RNA binding 2; *TI1*: trypsin inhibitor protein 1; *KTI1*: Kunitz trypsin inhibitor 1; *CYP71*: cytochrome P450 family 71; *PPXCB*: peroxidase CB. (D) Validation of the expression levels of five selected cluster 2 genes by qRT-PCR. *AZI1*: azelatic acid induced 1; *FRK1*: FLG22-induced receptor-like kinase 1; *LRR*: leucine-rich repeat protein kinase family protein; *JRG21*: jasmonate-regulated gene 21; *CRK13*: cysteine-rich RLK (RECEPTOR-like protein kinase) 13. Transcript levels of genes were normalized relative to those of *AtACT2* (GenBank accession no. Q96292).

To investigate whether *V*. *vulnificus* modulates *Arabidopsis* plants at the molecular level, we investigated gene expression levels in *Arabidopsis* leaves infiltrated with *V*. *vulnificus* 96-11-17M at 0, 12, 24, 48 and 72 h post-inoculation *in planta* analysis. The correlation coefficients of all samples were highly similar, suggesting that none of the samples exhibited abnormal or artificial variation in gene expression levels (S1 Fig). Unsupervised hierarchical clustering analysis revealed genes showing significantly different expression patterns at each time point compared with the 0 h time point (control) and classified the genes into four clusters (Fig 2A). According to the results of heat map and line plot analyses, cluster 1 genes were specifically up-regulated at 48 h post-inoculation, cluster 2 genes were up-regulated at both 12 and 24 h post-inoculation (relatively early time points), cluster 3 genes were down-regulated at tested time points compared with 0 h control and cluster 4 genes were down-regulated from 12 to 48 h post-inoculation (Fig 2B). To validate the RNA-Seq data, we examined the expression of five cluster 1 and cluster 2 genes using quantitative real-time PCR (qRT-PCR). The expression of these genes was relatively high at 48 h or from 12 to 24 h after *V*. *vulnificus* inoculation (Fig 2C and 2D).

**Fig 3:**
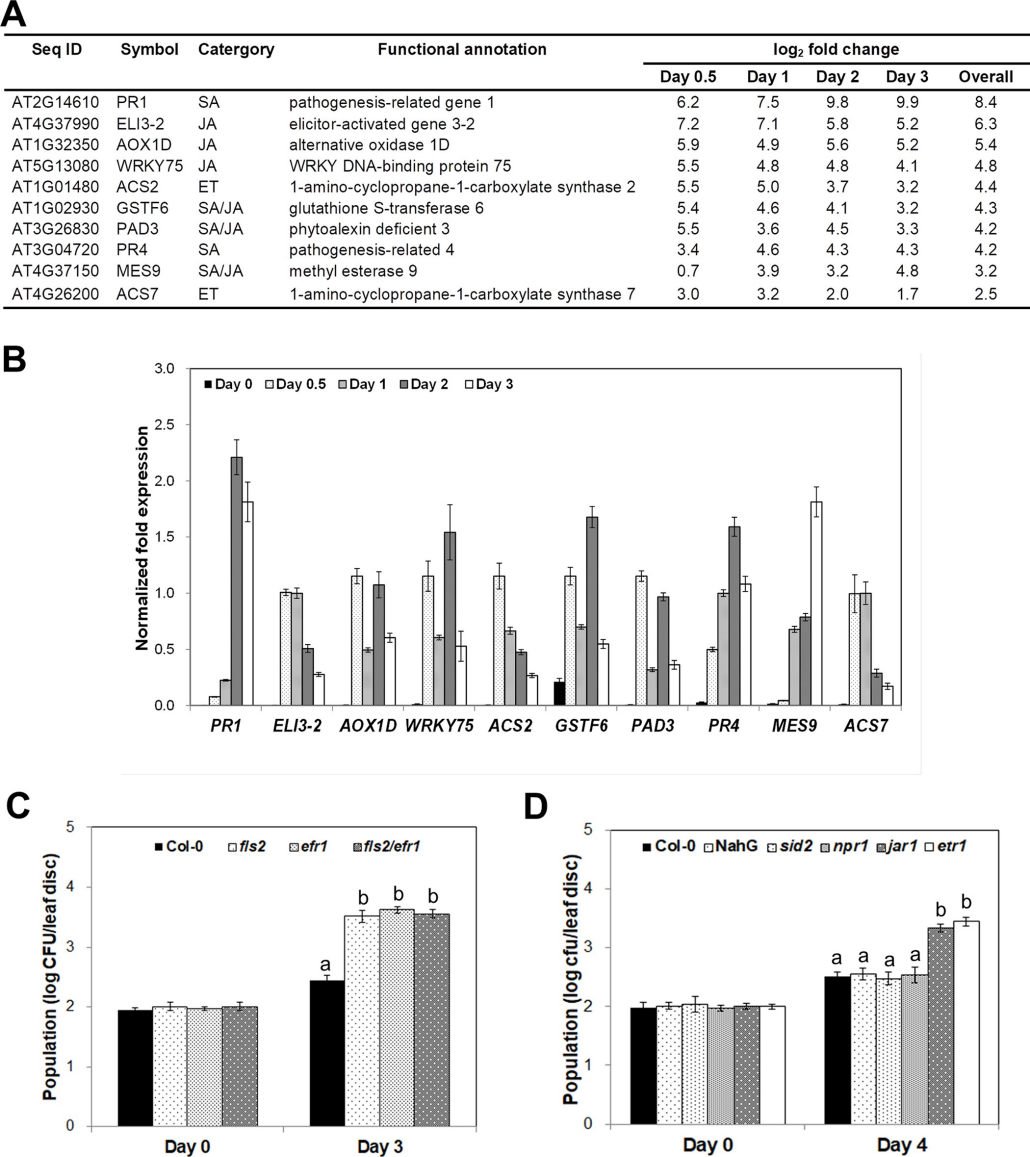
*V*. *vulnificus* 96-11-17M modulates plant defense responses. Expression levels of selected plant defense-related genes determined by RNA-Seq and qRT-PCR analyses are shown. The relative fold change in gene expression was calculated at each time point relative to the control time point. (A) Expression profiles of 10 defense-related genes in *Arabidopsis* plants inoculated with *V*. *vulnificus* 96-11-17M. Transcript levels of genes were quantified by RNA-Seq analysis. (B) Validation of the RNA-Seq data of 10 *Arabidopsis* genes by qRT-PCR. Transcript levels of genes were normalized relative to those of *AtACT2* (GenBank accession no. Q96292). (C,D) Total population density of *V*. *vulnificus* 96-11-17M in three pathogen-associated molecular pattern (PAMP) receptor mutants at 3 days post-inoculation (C) and phytohormone signaling mutants at 4 days post-inoculation (D). Different letters above the bars indicate significant differences (*P* = 0.05) between genotypes.

Next, we aimed to identify genes showing differential expression at specific time points compared with the 0 h time point (control). A total of 5,360, 4,204, 4,916 and 3,741 DEGs were identified at 12, 24, 48 and 72 h post-inoculation with *V*. *vulnificus* 96-11-17M, respectively, relative to the control (Table 1). Expression levels of the top 20 DEGs at each time point are listed in S2–S5 Tables. To elucidate the potential biological functions of these DEGs, we performed Gene Ontology (GO) analysis using the DAVID software. According to the GO biological process categories, 20 DEGs were potentially involved in plant defense-related hormones and reactive oxygen species (ROS) (S2 Fig). This result prompted us to identify DEGs related to SA-, JA- and ET-dependent signaling pathways; DEGs showing significant variation in expression at various time points are listed in S6 Table. We validated the expression levels of the top 10 up-regulated genes using qRT-PCR. The results showed that all 10 genes were significantly induced by *V*. *vulnificus* infiltration, and the fold change ratios were clearly comparable to the RNA-Seq data (Fig 3A and 3B).

**Table 1:** Number of differentially expressed genes (DEGs) in *Arabidopsis thaliana* plants inoculated with *V*. *vulnificus* 96-11-17M at the indicated time points.

Comparison	Number of DEGs*	Number of up-regulated DEGs	Number of down-regulated DEGs
0 h vs. 12 h	5,360	2,492	2,868
0 h vs. 24 h	4,204	2,075	2,129
0 h vs. 48 h	4,916	2,473	2,443
0 h vs. 72 h	3,741	1,851	1,890

*DEGs, gene showing at least 2-fold difference in expression between the control time point (0 h) and other time points.

### *V*. *vulnificus* is likely perceived by common receptors of *Arabidopsis* and activates JA and ET signaling

To investigate how *V*. *vulnificus* affects the plant defense mechanism for its propagation *in planta*, we monitored the population density of *V*. *vulnificus* in wild-type (Col-0) and mutant plants of *Arabidopsis*; these knockout mutants lacked genes that encode pattern recognition receptors (PRRs), such as Flagellin Sensing2 (FLS2) and Elongation Factor 2 Related 1 (EFR1), that recognize pathogen/microbe-associated molecular patterns (PAMPs/MAMPs). Higher *V*. *vulnificus* population densities were observed in the knockout mutant lines than in wild-type plants at 3 days post-infiltration (Fig 3C), indicating that PAMPs/MAMPs of *V*. *vulnificus* such as flagellin and elongation factor are recognized by common PRRs to activate plant innate immunity.

In addition, analyses of RNA-Seq data revealed that genes involved in plant defense hormone-related pathways were up-regulated in *V*. *vulnificus*-infiltrated plants (S2 Fig and S6 Table). These results suggested that defense-related hormone pathways should be required for *V*. *vulnificus* infiltration. This phenomenon needed to be confirmed by genetic analyses of *Arabidopsis*; we quantified the bacterial population density in wild-type and mutant *Arabidopsis* plants at 4 days post-infiltration *in planta*. The population density of *V*. *vulnificus* was the same in the SA signaling pathways NahG transgenic, SA-deficient mutant (*sid2*) and *Nonexpressor of Pathogenesis-Related gene 1* mutant (*npr1*) lines compared with the Col-0 (control). By contrast, the bacterial population density was at least 10-fold higher in the JA response mutant (*jar1*) and Ethylene Receptor 1 mutant (*etr1*) than in the Col-0 (control) (Fig 3D).

### *V*. *vulnificus* shares common features with *Pseudomonas syringae* pv. *maculicola* ES4326

**Fig 4:**
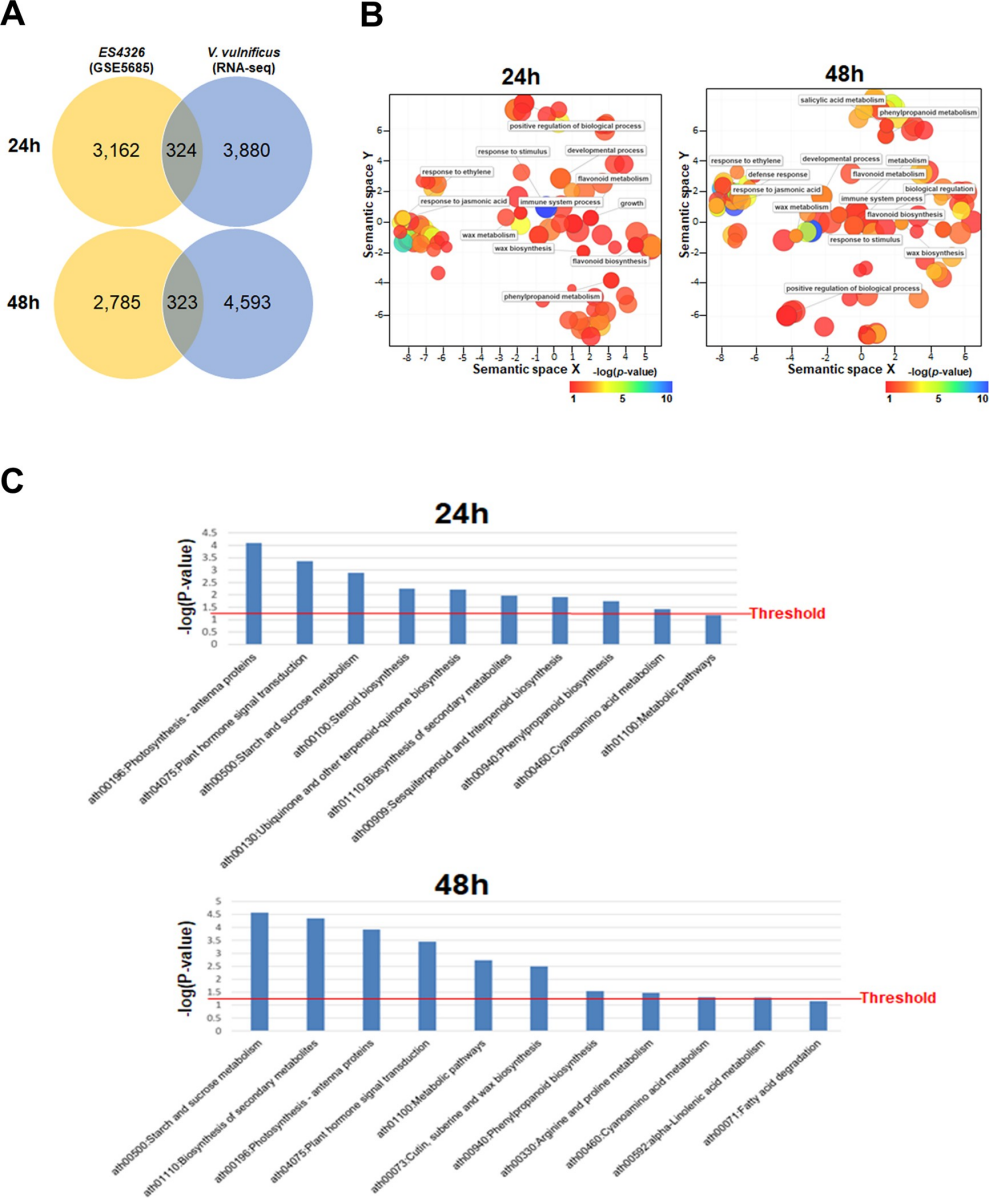
Comparative transcriptome analysis of *Arabidopsis* plants inoculated with the plant pathogen *P*. *syringae* pv. *maculicola* ES4326 and the marine pathogen *V*. *vulnificus* 96-11-17M. Transcriptome data of *Arabidopsis* plants inoculated with *P*. *syringae* were obtained from a publicly available database (GSE number: 5685). (A) Venn diagram showing the total number of differentially expressed genes (DEGs) in each transcriptome data set and the number of DEGs overlapping between the two data sets. (B) Bubble charts displaying Gene Ontology (GO) enrichment analysis of the overlapping genes. GO biological process category was applied. C: Bar charts displaying the results of Kyoto Encyclopaedia of Genes and Genomes (KEGG) pathway enrichment analysis of the overlapping genes.

Because our results suggested *V*. *vulnificus* modulated *Arabidopsis* defense-related genes, we compared the transcriptome data obtained from *Arabidopsis* plants inoculated with *V*. *vulnificus* with transcriptome data obtained from *Arabidopsis* plants inoculated with *P*. *syringae* pv. *maculicola* ES4326 (GSE 5685), a well known bacterial plant pathogen. This comparison revealed 3,486 and 3,108 DEGs in *Arabidopsis* at 24 and 48 h post-inoculation with *P*. *syringae* pv. *maculicola* ES4326, respectively (Fig 4A), of which 324 and 323 DEGs overlapped with those identified in *V*. *vulnificus* 96-11-17M-infiltrated *Arabidopsis* plants at 24 and 48 h, respectively (Fig 4A). Among overlapped genes, *PATHOGENESIS-RELATED 1* (*PR1*), *FLG22-INDUCED RECEPTOR-LIKE KINASE 1* (*FRK1*), *NITRATE TRANSPORTER 2*.*6* (*NRT2*.*6*), *SENESCENCE-ASSOCIATED GENE 13* (*SAG13*) and *ELICITOR PEPTIDE 3 PRECURSOR* (*PROPEP3*) were top-5 inducible genes in *Arabidopsis* at 24 h and *PATHOGENESIS-RELATED 1* (*PR1*), *CYTOCHROME P450 FAMILY 71 SUBFAMILY A POLYPEPTIDE 12* (*CYP71A12*), *CINNAMOYL COA REDUCTASE* (*CCR2*), *EARLY FLOWERING 4* (*EFL4*) and *FLG22-INDUCED RECEPTOR-LIKE KINASE 1* (*FRK1*) genes were listed in *Arabidopsis* at 48 h. The results of GO analysis showed that, under the biological process category, genes overlapping between the two transcriptome data sets were classified under response to JA, response to ET, response to stimulus, immune system process, flavonoid biosynthesis, flavonoid metabolism, phenylpropanoid metabolism, developmental process, and wax metabolism and biosynthesis, regardless of the sampling time point (Fig 4B). The results of Kyoto Encyclopaedia of Genes and Genomes (KEGG) pathway analysis showed that, in plants sampled at 24 and 48 h, many of these overlapping genes were involved in photosynthesis, plant hormone signal transduction and starch and sucrose metabolism (Fig 4C). The 324 and 323 *Arabidopsis* genes were significantly up- and down-regulated by both *V*. *vulnificus* 96-11-17M and *P*. *syringae* pv. *maculicola* ES4326 at 24 and 48 h post-infection, respectively. Between 324 and 323 DEGs at two time points, approximately 80% of genes were overlapped (S7 Table).

## Discussion

Despite the increasing interest in fresh food contamination and rising sea level, there has yet been no evidence suggesting that the marine bacterial pathogen *V*. *vulnificus* regulates the transcriptome and physiology of plants. In the current study, we showed that *V*. *vulnificus* infection caused disease symptoms in *Arabidopsis* plants, consistent with the increase in its population density under artificial and favorable conditions. Moreover, *V*. *vulnificus* modulated the transcriptome of *Arabidopsis* plants at certain time points, and a considerable overlap in DEGs was observed between *Arabidopsis* plants infiltrated with *V*. *vulnificus* and those infected with *P*. *syringae* pv. *maculicola* ES4326. These overlapping DEGs were further characterized to obtain potential functional clues that indicate how *V*. *vulnificus* modulates *Arabidopsis*.

In this study, we first asked how *V*. *vulnificus* could survive in plants and develop pathogenicity and virulence against plants under natural conditions. To maintain the pathogenicity and virulence of *V*. *vulnificus* in *Arabidopsis*, we artificially maintained the bacterial colonies under favorable conditions on LB agar medium containing 2% sodium chloride, with salinity ranging from 15 to 25 parts per thousand [26,27]. To inoculate and infiltrate plants, bacterial colonies were resuspended in 10% PBS, thus supplying additional NaCl. The resuspended bacterial colonies were subsequently infiltrated into the apoplast, which is the space between the plasma membrane and cell wall, to enable easy penetration into the plant cell by avoiding the cell wall. The results showed typical disease symptoms on *Arabidopsis* leaves both *in vitro* and *in planta*, accompanied by an increase in the population density of *V*. *vulnificus* in infected plants (Fig 1B). These results indicate that *V*. *vulnificus* acts normally in the plant host under favorable conditions.

To complete its life cycle under unfavorable conditions, *V*. *vulnificus* must overcome the immune response of the human body [28]. The plant cell apoplast represents a favorable habitat for pathogens, as it mediates the transport of water and solutes between tissues or organs and provides a carbon dioxide (CO_2_)-rich environment [29]. *V*. *vulnificus* uptakes nutrients and converts amino acids to amines and CO_2_ under acidic conditions [28,30]. The apoplast undergoes acidification in plant roots, coleoptiles, internodes and leaves [29]. Intriguingly, in this study, parameters such as acidification, salinity and CO_2_ levels of the plant apoplast were appropriate for facilitating the infection of *Arabidopsis* plants by *V*. *vulnificus*.

Next, it is important to explain how plants respond to the infection by *V*. *vulnificus*. The notion that *V*. *vulnificus* acts as a plant pathogen is based on the hypothesis that plants have evolved an elaborate immune system that activates defense pathways in response to *V*. *vulnificus* infection. The first line of plant defense is the activation of PRRs localized in the plasma membrane upon the recognition of PAMPs/MAMPs [31]. To investigate whether the common PAMP receptors FLS2 and EFR1in *Arabidopsis* are involved in the perception of flagellin and elongation factor, respectively, and thus in the activation of the plant defense response, we quantified the population density of *V*. *vulnificus* in wild-type and PAMP receptor mutant plants of *Arabidopsis*. The bacterial population density was 10-fold higher in the mutants than in wild-type plants (Fig 3C). These results indicate that the human pathogen *V*. *vulnificus* is recognized by plant receptors and acts as a pathogen in *Arabidopsis* under favorable conditions.

The plant hormones SA, JA and ET play a pivotal role in plant defense against pathogens. Biotrophic pathogens that activate programmed cell death in plants are dependent on SA signal transduction, whereas necrotrophic pathogens rely on JA and ET signaling pathways [32]. In agreement with the above notion, genes involved in SA, JA and ET signaling pathways were highly up-regulated in *V*. *vulnificus*-infiltrated plants at all time points (Fig 3A and 3B and Fig 4). This suggests that *V*. *vulnificus* modulates plant defense-related hormone signaling pathways. This phenomenon was confirmed by genetic analyses of *Arabidopsis jar1* and *etr1* mutants showing that the bacterial population density was higher in these mutants than in the wild type (Col-0; Fig 3D). Thus, gene expression analysis and genetic analyses indicate that *V*. *vulnificus* modulates plant defense response through JA- and ET-dependent signaling pathways.

**Fig 5:**
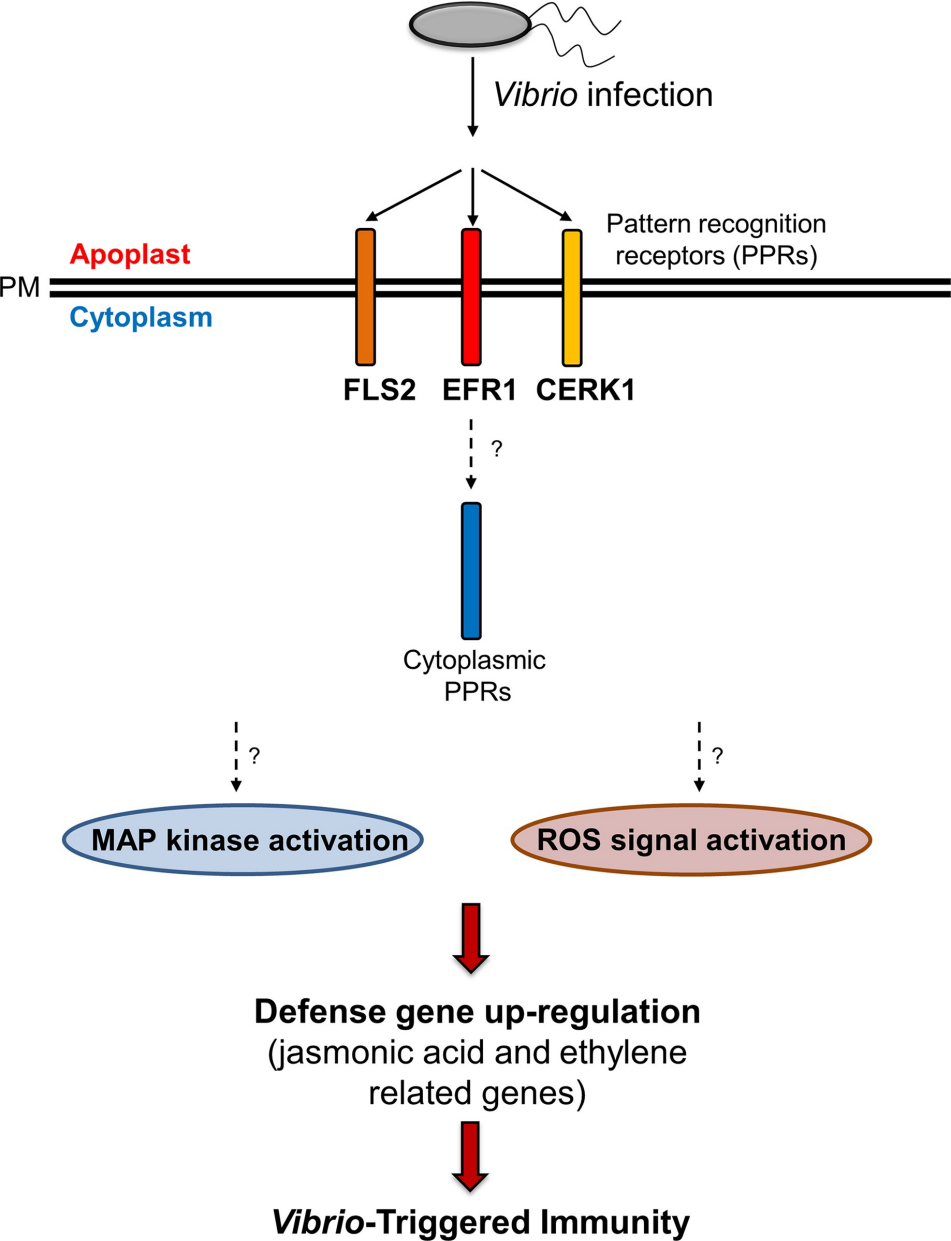
Schematic representation of the model showing the *Arabidopsis* and *V*. *vulnificus* interaction. Infection by *V*. *vulnificus* is perceived by common plant pattern recognition receptors (PRRs) such as FLS2 and EFR1, and this signal may be transferred to the intracellular PRRs. In the plant cell, *V*. *vulnificus* infection activates the MAP kinase signaling cascade and reactive oxygen species (ROS). This up-regulates the expression of defense-related genes, resulting in the elicitation of *V*. *vulnificus*-triggered immunity. PM: plasma membrane.

In conclusion, *V*. *vulnificus* clearly produced disease symptoms in *Arabidopsis* leaves both *in vitro* and *in planta* under artificial and favorable conditions. We propose that *V*. *vulnificus* infects plants by activating common PRRs and regulates defense-related gene expression and plant defense responses (Fig 5). Our results revealed several intriguing aspects of how *V*. *vulnificus* to regulate *Arabidopsis* under favorable conditions. Here, we describe for the first time the possible mechanism of infection of *Arabidopsis* by *V*. *vulnificus*, thus expanding the current knowledge on the pathogenicity of *V*. *vulnificus*. If we verify the communication between *V*. *vulnificus* and *A*. *thaliana* under natural conditions in further investigation it should prompt public health officials to pay more attention to the contamination of fresh vegetables and crops by *V*. *vulnificus*, which may be as important as infection with *Salmonella* and *E*. *coli* O157. In addition, the evidence provided in this study contributes to the screening and elucidation of virulence factors of *V*. *vulnificus* alternatively using plant systems, which can ultimately be used to eradicate animal and human diseases caused by *V*. *vulnificus*. Taken together, our results reveal a new paradigm that a human pathogenic marine bacterium modulates defense responses in *Arabidopsis* under artificial and favorable conditions.

## Supporting information

S1 Fig Scatterplot matrices of RNA-sequencing data in 10 *Arabidopsis* samples. Scatter plots with Lowess line between all pairs of samples were illustrated at the left lower boxes. Correlation coefficients between samples were described in the right upper boxes. Each box located at diagonal line displayed sample ID. ARTH, *Arabidopsis thaliana*.
(TIF)

S2 Fig Gene Ontology (GO) analysis of differentially expressed genes (DEGs) after *V. vulnificus* 96-11-17M inoculation. GO categories were assigned for DEGs from *Arabidopsis* at 12, 24, 48 and 72 h after *V. vulnificus* 96-11-17M infiltration compared to the control (0 h) using DAVID software.
(TIF)

S1 Table Primer list used in this study. (DOCX)

S2 Table Top 20 genes of DEGs at 12 h after *V. vulnificus* 96-11-17M infiltration. (DOCX)

S3 Table Top 20 genes of DEGs at 24 h after *V. vulnificus* 96-11-17M infiltration. (DOCX)

S4 Table Top 20 genes of DEGs at 48 h after *V. vulnificus* 96-11-17M infiltration. (DOCX)

S5 Table Top 20 genes of DEGs at 72 h after *V. vulnificus* 96-11-17M infiltration. (DOCX)

S6 Table Expression of defense-related genes. (DOCX)

S7 Table Overlapped DEGs between *P. syringae* and *V. vulnificus* 96-11-17M infiltration. (DOCX)
