# Supplementary figures and images for: The transcriptome analysis of the Arabidopsis thaliana in response to the Vibrio vulnificus by RNA-sequencing
Source: PLoS One. 2019 Dec 16;14(12):e0225976. doi: 10.1371/journal.pone.0225976 (PMC6913959; doi:10.1371/journal.pone.0225976)

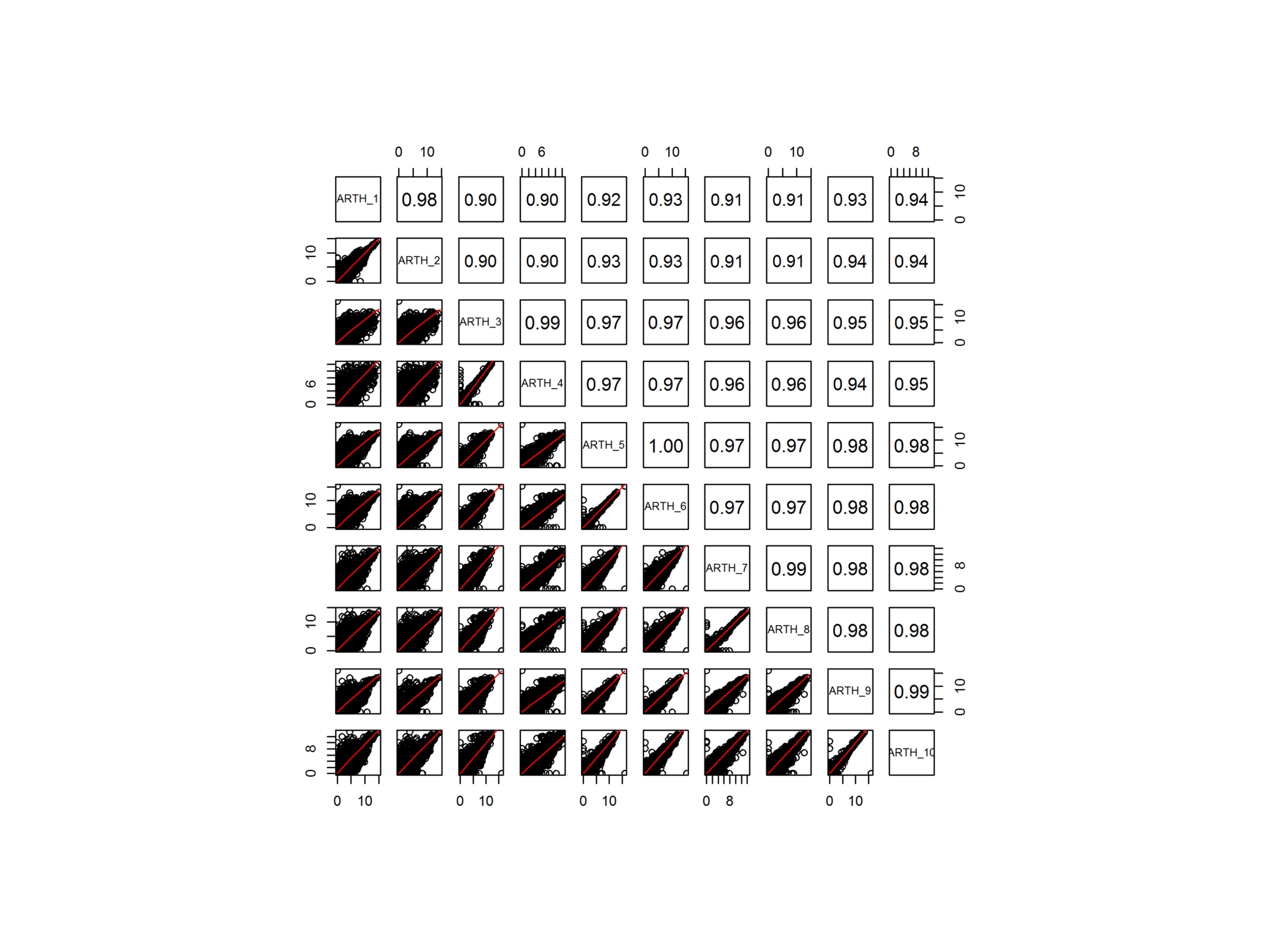

Supplement: S1 Fig — Scatter plots with Lowess line between all pairs of samples were illustrated at the left lower boxes. Correlation coefficients between samples were described in the right upper boxes. Each box located at diagonal line displayed sample ID. ARTH, Arabidopsis thaliana. (TIF) [file pone.0225976.s001.TIF]

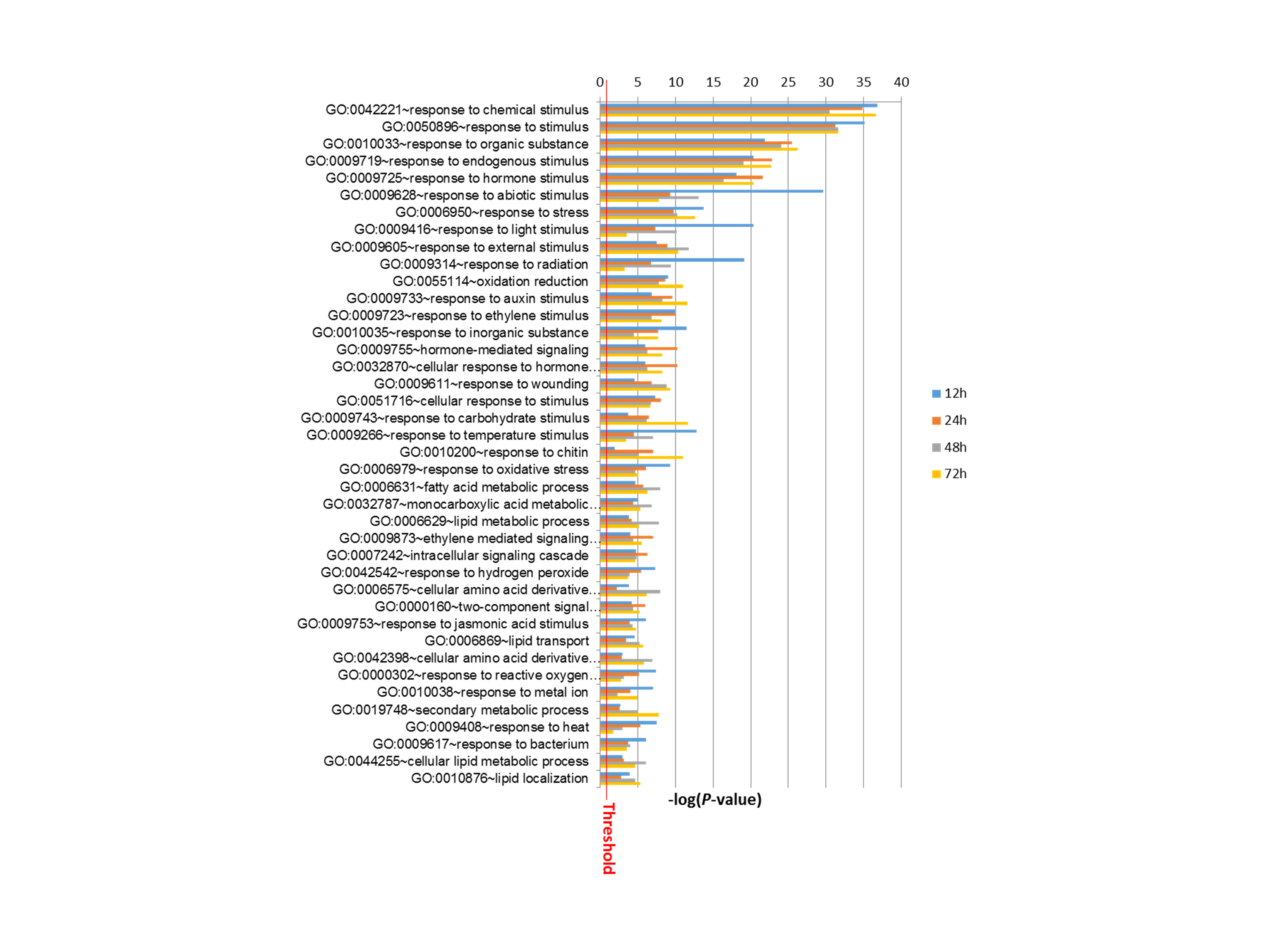

Supplement: S2 Fig — GO categories were assigned for DEGs from Arabidopsis at 12, 24, 48 and 72 h after V. vulnificus 96-11-17M infiltration compared to the control (0 h) using DAVID software. (TIF) [file pone.0225976.s002.TIF]
