# Supplementary material for: The transcriptome analysis of the Arabidopsis thaliana in response to the Vibrio vulnificus by RNA-sequencing
Source: PLoS One. 2019 Dec 16;14(12):e0225976. doi: 10.1371/journal.pone.0225976 (PMC6913959; doi:10.1371/journal.pone.0225976)
Supplement: S1 Table — (DOCX) [file pone.0225976.s003.docx]

**S1Table.** Primer list used in this study.
