# Supplementary material for: The transcriptome analysis of the Arabidopsis thaliana in response to the Vibrio vulnificus by RNA-sequencing
Source: PLoS One. 2019 Dec 16;14(12):e0225976. doi: 10.1371/journal.pone.0225976 (PMC6913959; doi:10.1371/journal.pone.0225976)
Supplement: S2 Table — (DOCX) [file pone.0225976.s004.docx]

**S2Table.** Top 20 genes of DEGs at 12 h after *V. vulnificus* 96-11-17M infiltration.

| **Gene Symbol** | **0h-1** | **0h-2** | **12h-1** | **12h-2** | **Fold change**  **(log_2_ ratio, 12h/0h)** |
| --- | --- | --- | --- | --- | --- |
| AT4G12500 | 2.637 | 2.022 | 10.745 | 11.063 | 8.575 |
| AZI1 | 3.383 | 2.284 | 10.915 | 11.188 | 8.218 |
| pEARLI 1 | 3.736 | 2.942 | 11.228 | 11.485 | 8.017 |
| AT4G12490 | 4.435 | 3.905 | 11.981 | 12.337 | 7.989 |
| AT2G39518 | 0.721 | 1.425 | 8.783 | 8.951 | 7.795 |
| AT4G22470 | 1.820 | 1.490 | 9.438 | 9.371 | 7.750 |
| AT1G53540 | 0.178 | 0.456 | 7.906 | 7.903 | 7.588 |
| AT2G43620 | 2.837 | 3.568 | 10.580 | 10.995 | 7.585 |
| AT3G18250 | 1.960 | 1.048 | 8.913 | 9.117 | 7.511 |
| AT2G45220 | 1.203 | 0.620 | 8.473 | 8.228 | 7.439 |
| AT2G10940 | 10.803 | 10.514 | 4.145 | 5.025 | -6.073 |
| AT1G15825 | 7.365 | 7.060 | 1.069 | 1.046 | -6.155 |
| LHCB4.2 | 13.009 | 12.394 | 6.475 | 6.587 | -6.171 |
| CAB3 | 12.481 | 11.926 | 5.160 | 5.900 | -6.673 |
| LHCB3 | 12.850 | 12.455 | 5.791 | 6.020 | -6.747 |
| CAB2 | 13.260 | 12.784 | 6.073 | 6.368 | -6.802 |
| LHCB2.1 | 12.624 | 12.418 | 5.241 | 5.753 | -7.024 |
| AT1G32900 | 8.511 | 9.314 | 1.699 | 1.693 | -7.216 |
| LHCB2.2 | 12.029 | 11.847 | 4.020 | 4.378 | -7.739 |
| LHCB2.3 | 10.424 | 9.688 | 2.026 | 2.017 | -8.034 |
| 1. The red and green colors indicated the up- and down-regulation, respectively.  2. Ten up-regulated and 10 down-regulated genes were listed. | | | | | |
