# Supplementary material for: The transcriptome analysis of the Arabidopsis thaliana in response to the Vibrio vulnificus by RNA-sequencing
Source: PLoS One. 2019 Dec 16;14(12):e0225976. doi: 10.1371/journal.pone.0225976 (PMC6913959; doi:10.1371/journal.pone.0225976)
Supplement: S3 Table — (DOCX) [file pone.0225976.s005.docx]

**S3 Table.** Top 20 genes of DEGs at 24 h after *V. vulnificus* 96-11-17M infiltration.

| **Gene Symbol** | **0h-1** | **0h-2** | **24h-1** | **24h-2** | **Fold change**  **(log_2_ ratio, 24h/0h)** |
| --- | --- | --- | --- | --- | --- |
| AT2G01021 | 0.000 | 0.000 | 16.035 | 15.356 | 15.696 |
| AT4G12490 | 4.435 | 3.905 | 12.885 | 12.831 | 8.688 |
| AT4G12500 | 2.637 | 2.022 | 10.905 | 10.860 | 8.553 |
| pEARLI 1 | 3.736 | 2.942 | 11.269 | 11.315 | 7.953 |
| AT5G43580 | 1.187 | 0.250 | 8.435 | 8.866 | 7.932 |
| AZI1 | 3.383 | 2.284 | 10.743 | 10.637 | 7.857 |
| AT5G44575 | 2.589 | 1.477 | 9.934 | 9.723 | 7.795 |
| AT4G22470 | 1.820 | 1.490 | 9.627 | 9.267 | 7.792 |
| EXT4 | 4.807 | 3.753 | 12.023 | 11.940 | 7.701 |
| AT2G45220 | 1.203 | 0.620 | 8.599 | 8.477 | 7.626 |
| AT1G04800 | 5.779 | 5.537 | 1.282 | 1.648 | -4.193 |
| AT5G22920 | 7.468 | 6.237 | 2.405 | 2.901 | -4.200 |
| BZIP61 | 5.161 | 4.720 | 0.668 | 0.737 | -4.238 |
| AT3G62950 | 6.614 | 6.493 | 2.221 | 2.330 | -4.278 |
| AT1G29670 | 8.265 | 8.307 | 3.866 | 4.055 | -4.326 |
| AT3G06070 | 9.734 | 9.412 | 4.758 | 5.023 | -4.682 |
| AT5G28630 | 7.267 | 6.580 | 2.211 | 2.207 | -4.714 |
| EXPA16 | 5.347 | 5.508 | 0.451 | 0.610 | -4.897 |
| BXL2 | 6.933 | 4.790 | 0.674 | 0.887 | -5.081 |
| AT1G72416 | 9.284 | 7.732 | 3.589 | 3.245 | -5.091 |
| 1. The red and green colors indicated the up- and down-regulation, respectively.  2. Ten up-regulated and 10 down-regulated genes were listed. | | | | | |
