# Supplementary material for: The transcriptome analysis of the Arabidopsis thaliana in response to the Vibrio vulnificus by RNA-sequencing
Source: PLoS One. 2019 Dec 16;14(12):e0225976. doi: 10.1371/journal.pone.0225976 (PMC6913959; doi:10.1371/journal.pone.0225976)
Supplement: S4 Table — (DOCX) [file pone.0225976.s006.docx]

**S4 Table.** Top 20 genes of DEGs at 48 h after *V. vulnificus* 96-11-17M infiltrarion.

| **Gene Symbol** | **0h-1** | **0h-2** | **48h-1** | **48h-2** | **Fold change**  **(log_2_ ratio, 48h/0h)** |
| --- | --- | --- | --- | --- | --- |
| AT4G12490 | 4.435 | 3.905 | 14.270 | 14.428 | 10.179 |
| PR1 | 1.747 | 2.452 | 11.963 | 11.834 | 9.799 |
| AT4G12500 | 2.637 | 2.022 | 11.561 | 11.616 | 9.260 |
| pEARLI 1 | 3.736 | 2.942 | 12.446 | 12.413 | 9.090 |
| AT3G18250 | 1.960 | 1.048 | 10.374 | 10.006 | 8.686 |
| AZI1 | 3.383 | 2.284 | 10.751 | 10.812 | 7.948 |
| EXT4 | 4.807 | 3.753 | 12.308 | 11.801 | 7.774 |
| TI1 | 3.940 | 3.376 | 11.340 | 11.260 | 7.642 |
| KTI1 | 1.176 | 0.782 | 8.390 | 8.489 | 7.461 |
| AT5G44575 | 2.589 | 1.477 | 9.562 | 9.395 | 7.445 |
| AT3G06070 | 9.734 | 9.412 | 4.644 | 4.299 | -5.102 |
| AT4G21870 | 8.755 | 7.369 | 3.026 | 2.594 | -5.252 |
| BETA-OHASE 2 | 6.308 | 7.206 | 1.394 | 1.522 | -5.299 |
| EXPA16 | 5.347 | 5.508 | 0.000 | 0.095 | -5.380 |
| AT1G32900 | 8.511 | 9.314 | 3.554 | 3.038 | -5.617 |
| AT5G28630 | 7.267 | 6.580 | 1.151 | 1.279 | -5.708 |
| AT1G21910 | 9.178 | 8.077 | 2.934 | 2.603 | -5.858 |
| PHI-1 | 8.874 | 8.114 | 2.567 | 2.203 | -6.109 |
| EXO | 8.151 | 8.386 | 1.999 | 2.135 | -6.201 |
| LHY | 7.580 | 7.608 | 1.041 | 1.016 | -6.566 |
| 1. The red and green colors indicated the up- and down-regulation, respectively.  2. Ten up-regulated and 10 down-regulated genes were listed. | | | | | |
