# Supplementary material for: The transcriptome analysis of the Arabidopsis thaliana in response to the Vibrio vulnificus by RNA-sequencing
Source: PLoS One. 2019 Dec 16;14(12):e0225976. doi: 10.1371/journal.pone.0225976 (PMC6913959; doi:10.1371/journal.pone.0225976)
Supplement: S5 Table — (DOCX) [file pone.0225976.s007.docx]

**S5 Table.** Top 20 genes of DEGs at 72 h after *V. vulnificus* 96-11-17M infiltrarion.

| **Gene Symbol** | **0h-1** | **0h-2** | **72h-1** | **72h-2** | **Fold change**  **(log_2_ ratio, 72h/0h)** |
| --- | --- | --- | --- | --- | --- |
| PR1 | 1.747 | 2.452 | 11.941 | 12.015 | 9.879 |
| MT1A | 0.000 | 0.000 | 8.816 | 10.026 | 9.421 |
| AT4G12490 | 4.435 | 3.905 | 13.141 | 13.401 | 9.101 |
| AT4G12500 | 2.637 | 2.022 | 10.294 | 10.468 | 8.051 |
| KTI1 | 1.176 | 0.782 | 8.946 | 8.931 | 7.960 |
| pEARLI 1 | 3.736 | 2.942 | 11.084 | 11.154 | 7.780 |
| AT3G18250 | 1.960 | 1.048 | 9.075 | 9.411 | 7.739 |
| TI1 | 3.940 | 3.376 | 10.900 | 11.056 | 7.320 |
| EXT4 | 4.807 | 3.753 | 11.441 | 11.705 | 7.293 |
| AT5G43580 | 1.187 | 0.250 | 7.691 | 8.157 | 7.206 |
| AT1G04800 | 5.779 | 5.537 | 1.208 | 1.525 | -4.291 |
| AT5G22580 | 8.095 | 7.735 | 3.278 | 3.808 | -4.372 |
| STP1 | 10.054 | 9.861 | 5.266 | 5.847 | -4.401 |
| AT4G21870 | 8.755 | 7.369 | 3.264 | 3.631 | -4.614 |
| BXL2 | 6.933 | 4.790 | 0.981 | 1.332 | -4.705 |
| AT3G44260 | 9.787 | 7.636 | 4.034 | 3.802 | -4.794 |
| AT1G72416 | 9.284 | 7.732 | 3.572 | 3.752 | -4.846 |
| EXPA16 | 5.347 | 5.508 | 0.345 | 0.777 | -4.867 |
| AT3G06070 | 9.734 | 9.412 | 4.431 | 4.880 | -4.918 |
| AT5G28630 | 7.267 | 6.580 | 0.998 | 1.237 | -5.805 |
| 1. The red and green colors indicated the up- and down-regulation, respectively.  2. Ten up-regulated and 10 down-regulated genes were listed. | | | | | |
