# Supplementary material for: The transcriptome analysis of the Arabidopsis thaliana in response to the Vibrio vulnificus by RNA-sequencing
Source: PLoS One. 2019 Dec 16;14(12):e0225976. doi: 10.1371/journal.pone.0225976 (PMC6913959; doi:10.1371/journal.pone.0225976)
Supplement: S6 Table — (DOCX) [file pone.0225976.s008.docx]

**S6Table.** Expression of defense-related genes.

| **Pathway Gene** | **Pathway Symbol** | **Pathway Type** | **Pathway Name** | **Fold change log ratio (12h/0h)** | **Fold change log ratio (24h/0h)** | **Fold change log ratio (48h/0h)** | **Fold change log ratio (72h/0h)** |
| --- | --- | --- | --- | --- | --- | --- | --- |
| AT2G14610 | PR1 | SA | SA response | 6.241 | 7.536 | 9.799 | 9.879 |
| AT4G37990 | ELI3-2 | JA | oxylipin response | 7.200 | 7.130 | 5.764 | 5.247 |
| AT1G32350 | AOX1D | JA | oxylipin response | 5.877 | 4.923 | 5.629 | 5.228 |
| AT5G13080 | WRKY75 | JA | oxylipin response | 5.451 | 4.775 | 4.766 | 4.088 |
| AT1G01480 | ACS2 | ET | ET synthesis | 5.523 | 4.991 | 3.716 | 3.227 |
| AT1G02930 | GSTF6 | JA | JA response | 5.369 | 4.572 | 4.080 | 3.157 |
| AT1G02930 | GSTF6 | SA | SA response | 5.369 | 4.572 | 4.080 | 3.157 |
| AT3G26830 | PAD3 | JA | oxylipin response | 5.482 | 3.558 | 4.522 | 3.315 |
| AT3G26830 | PAD3 | SA | SA response | 5.482 | 3.558 | 4.522 | 3.315 |
| AT3G04720 | PR4 | JA | JA response | 3.432 | 4.631 | 4.308 | 4.268 |
| AT3G04720 | PR4 | SA | SA response | 3.432 | 4.631 | 4.308 | 4.268 |
| AT3G63380 | AT3G63380 | JA | oxylipin response | 5.001 | 4.162 | 4.257 | 2.754 |
| AT5G37600 | GSR 1 | JA | JA response | 4.354 | 3.360 | 4.324 | 3.296 |
| AT1G02930 | GSTF6 | JA | JA response | 5.259 | 4.197 | 3.332 | 2.356 |
| AT1G02930 | GSTF6 | SA | SA response | 5.259 | 4.197 | 3.332 | 2.356 |
| AT5G02780 | GSTL1 | JA | oxylipin response | 3.811 | 2.920 | 3.540 | 3.344 |
| AT5G13320 | PBS3 | SA | aa conjugation of SA | 4.077 | 4.068 | 2.794 | 2.548 |
| AT4G37150 | MES9 | JA | JA synthesis | 0.724 | 3.942 | 3.179 | 4.826 |
| AT4G37150 | MES9 | SA | SA synthesis | 0.724 | 3.942 | 3.179 | 4.826 |
| AT2G37770 | AT2G37770 | JA | oxylipin response | 3.185 | 3.795 | 2.618 | 2.315 |
| AT2G29500 | AT2G29500 | JA | oxylipin response | 5.473 | 2.867 | 1.746 | 0.863 |
| AT4G26200 | ACS7 | ET | ET synthesis | 3.040 | 3.229 | 2.002 | 1.693 |
| AT5G22300 | NIT4 | JA | oxylipin response | 1.982 | 2.744 | 1.850 | 2.247 |
| AT3G05630 | PLDP2 | SA | link to lipid signaling | 2.252 | 2.516 | 2.009 | 2.037 |
| AT4G39950 | CYP79B2 | JA | JA response | 2.163 | 1.721 | 2.582 | 1.980 |
| AT2G25450 | AT2G25450 | JA | JA response | 1.262 | 2.304 | 2.175 | 2.300 |
| AT1G76790 | AT1G76790 | JA | JA response | 2.341 | 0.926 | 3.408 | 0.895 |
| AT1G09420 | G6PD4 | SA | SA signaling | 1.844 | 2.503 | 1.635 | 1.373 |
| AT3G12500 | HCHIB | JA | JA response | 1.279 | 1.933 | 1.877 | 2.174 |
| AT1G09240 | NAS3 | JA | JA response | -0.048 | 2.217 | 2.183 | 2.567 |
| AT1G78380 | GSTU19 | JA | JA response | 1.952 | 1.731 | 1.528 | 1.109 |
| AT2G37040 | PAL1 | JA | JA response | 1.269 | 1.895 | 1.296 | 1.718 |
| AT2G37040 | PAL1 | SA | SA synthesis | 1.269 | 1.895 | 1.296 | 1.718 |
| AT5G22860 | AT5G22860 | JA | oxylipin response | 1.393 | 1.961 | 1.598 | 1.172 |
| AT4G29010 | AIM1 | JA | JA synthesis | 1.275 | 1.513 | 1.617 | 1.456 |
| AT5G54810 | TSB1 | JA | JA response | 1.935 | 1.115 | 1.598 | 0.785 |
| AT5G14780 | FDH | JA | JA response | 1.183 | 2.020 | 0.924 | 1.205 |
| AT2G24850 | TAT3 | JA | JA response | 2.963 | 1.608 | 0.561 | -0.056 |
| AT3G12580 | HSP70 | JA | oxylipin response | 2.788 | 1.940 | -0.400 | 0.740 |
| AT3G57260 | BGL2 | SA | SA response | 1.931 | 0.204 | 1.154 | 1.676 |
| AT2G12190 | AT2G12190 | JA | oxylipin response | 0.272 | 1.206 | 1.534 | 1.678 |
| AT1G45145 | TRX5 | SA | SA signaling | 1.665 | 1.093 | 1.132 | 0.650 |
| AT2G23620 | MES1 | SA | SA synthesis | 0.162 | 1.140 | 1.183 | 1.976 |
| AT2G37770 | AT2G37770 | JA | oxylipin response | 1.256 | 1.708 | 0.777 | 0.283 |
| AT2G36880 | MAT3 | ET | ET synthesis | 0.770 | 1.337 | 0.518 | 1.225 |
| AT1G75040 | PR5 | SA | SA response | 1.725 | 0.051 | 1.031 | 0.911 |
| AT3G04580 | EIN4 | ET | ET signaling | 1.284 | 1.238 | 0.272 | 0.846 |
| AT4G00240 | PLDBETA2 | SA | link to lipid signaling | 1.272 | 0.800 | 1.079 | 0.351 |
| AT1G14410 | WHY1 | SA | SA signaling | 0.925 | 0.791 | 0.905 | 0.830 |
| AT5G06320 | NHL3 | SA | SA response | 1.070 | 0.256 | 1.289 | 0.770 |
| AT5G22860 | AT5G22860 | JA | oxylipin response | 0.656 | 0.394 | 1.398 | 0.922 |
| AT1G05180 | AXR1 | JA | JA signaling | 1.005 | 0.701 | 0.979 | 0.629 |
| AT5G14250 | COP13 | JA | JA signaling | 0.947 | 0.769 | 0.867 | 0.699 |
| AT2G03760 | SOT12 | SA | SA response | 1.724 | 0.919 | 0.468 | 0.006 |
| AT5G10300 | MES5 | SA | SA synthesis | 0.608 | 0.898 | 0.681 | 0.891 |
| AT4G11850 | PLDGAMMA1 | SA | link to lipid signaling | 1.077 | 0.632 | 1.008 | 0.193 |
| AT4G11850 | PLDGAMMA1 | SA | SA response | 1.077 | 0.632 | 1.008 | 0.193 |
| AT4G31500 | CYP83B1 | JA | JA response | 1.276 | 0.800 | 0.927 | -0.125 |
| AT4G11840 | PLDGAMMA3 | SA | link to lipid signaling | 0.744 | 0.883 | 0.894 | 0.303 |
| AT3G11480 | BSMT1 | SA | SA synthesis | 0.728 | 2.465 | 0.070 | -0.494 |
| AT5G05730 | ASA1 | JA | JA response | 1.606 | 0.437 | 0.723 | -0.202 |
| AT5G05730 | ASA1 | JA | JA response | 1.365 | 0.588 | 0.837 | -0.260 |
| AT1G55020 | LOX1 | JA | JA response | 0.287 | 0.605 | 0.390 | 0.984 |
| AT1G55020 | LOX1 | JA | JA synthesis | 0.287 | 0.605 | 0.390 | 0.984 |
| AT4G23810 | WRKY53 | SA | SA signaling | 0.863 | 0.897 | 0.553 | -0.068 |
| AT3G45140 | LOX2 | JA | JA response | -0.264 | 0.522 | 0.991 | 0.740 |
| AT3G45140 | LOX2 | JA | JA synthesis | -0.264 | 0.522 | 0.991 | 0.740 |
| AT4G23600 | CORI3 | JA | JA response | 0.646 | 0.360 | 0.741 | 0.234 |
| AT2G36880 | MAT3 | ET | ET synthesis | 0.297 | 0.551 | 0.739 | 0.379 |
| AT1G02500 | SAM1 | ET | ET synthesis | 0.519 | 0.243 | 0.407 | 0.723 |
| AT2G46370 | JAR1 | JA | aa conjugation of JA | 0.878 | 0.362 | 0.335 | 0.270 |
| AT4G16760 | ACX1 | JA | JA synthesis | 1.100 | 0.801 | -0.091 | -0.054 |
| AT5G03730 | CTR1 | ET | ET signaling | 0.794 | 0.243 | 0.441 | 0.260 |
| AT4G02570 | CUL1 | JA | JA signaling | 0.467 | 0.285 | 0.549 | 0.397 |
| AT5G20570 | RBX1 | JA | JA signaling | 0.399 | 0.522 | 0.432 | 0.307 |
| AT3G52400 | SYP122 | JA | JA signaling | 0.826 | 0.131 | 0.708 | -0.123 |
| AT3G52400 | SYP122 | SA | SA signaling | 0.826 | 0.131 | 0.708 | -0.123 |
| AT5G14250 | COP13 | JA | JA signaling | 0.127 | 0.574 | 0.108 | 0.617 |
| AT4G02570 | CUL1 | JA | JA signaling | 0.407 | 0.325 | 0.253 | 0.287 |
| AT4G02570 | CUL1 | JA | JA signaling | 0.633 | 0.033 | 0.377 | 0.215 |
| AT1G02500 | SAM1 | ET | ET synthesis | 0.291 | 0.024 | 0.234 | 0.662 |
| AT2G32950 | COP1 | JA | JA signaling | -0.328 | 0.596 | 0.158 | 0.738 |
| AT4G02570 | CUL1 | JA | JA signaling | 0.235 | 0.160 | 0.363 | 0.201 |
| AT1G76690 | OPR2 | JA | JA synthesis | 0.882 | -0.435 | 0.380 | 0.093 |
| AT1G64900 | CYP89A2 | JA | JA response | -1.953 | 0.712 | 0.928 | 1.146 |
| AT1G73730 | EIL3 | ET | ET signaling | 0.249 | 0.346 | 0.036 | 0.178 |
| AT1G05180 | AXR1 | JA | JA signaling | 0.000 | 0.355 | 0.098 | 0.313 |
| AT1G01480 | ACS2 | ET | ET synthesis | 0.144 | 0.590 | 0.000 | 0.000 |
| AT5G02780 | GSTL1 | JA | oxylipin response | 0.256 | 0.129 | 0.128 | 0.220 |
| AT1G66340 | ETR1 | ET | ET signaling | 0.089 | 0.290 | 0.087 | 0.108 |
| AT1G62380 | ACO2 | ET | ET synthesis | -0.286 | 1.096 | -0.431 | 0.194 |
| AT4G23600 | CORI3 | JA | JA response | 0.212 | 0.061 | -0.533 | 0.766 |
| AT1G54490 | XRN4 | ET | ET signaling | 0.171 | 0.060 | 0.212 | -0.051 |
| AT3G16785 | PLDP1 | SA | link to lipid signaling | 0.426 | -0.038 | 0.118 | -0.118 |
| AT3G23150 | ETR2 | ET | ET signaling | 0.419 | 0.195 | -0.119 | -0.167 |
| AT2G42010 | PLDBETA1 | SA | link to lipid signaling | 0.161 | -0.033 | -0.006 | 0.158 |
| AT1G52400 | BGLU18 | JA | JA response | 0.174 | -0.026 | 0.616 | -0.530 |
| AT4G23600 | CORI3 | JA | JA response | -0.105 | -0.110 | -0.263 | 0.705 |
| AT1G52400 | BGLU18 | JA | JA response | 0.226 | 0.000 | 0.000 | 0.000 |
| AT1G09420 | G6PD4 | SA | SA signaling | 0.159 | 0.010 | 0.027 | 0.007 |
| AT2G40940 | ERS1 | ET | ET signaling | 0.927 | -0.349 | 0.169 | -0.588 |
| AT3G52430 | PAD4 | SA | SA response | 0.524 | -0.035 | 0.064 | -0.406 |
| AT3G52430 | PAD4 | SA | SA synthesis | 0.524 | -0.035 | 0.064 | -0.406 |
| AT5G21120 | EIL2 | ET | ET signaling | 0.021 | 0.000 | 0.028 | 0.000 |
| AT1G13280 | AOC4 | JA | JA synthesis | -0.439 | -0.293 | 0.370 | 0.369 |
| AT4G01850 | SAM-2 | ET | ET synthesis | -0.220 | -0.073 | 0.517 | -0.223 |
| AT3G49700 | ACS9 | ET | ET synthesis | 0.000 | 0.000 | 0.000 | 0.000 |
| AT5G20570 | RBX1 | JA | JA signaling | 0.000 | 0.000 | 0.000 | 0.000 |
| AT5G51500 | AT5G51500 | JA | oxylipin response | 0.000 | 0.000 | 0.000 | 0.000 |
| AT1G24100 | UGT74B1 | JA | JA response | -0.110 | -0.147 | 0.129 | 0.112 |
| AT2G19590 | ACO1 | ET | ET synthesis | -0.014 | -0.067 | 0.122 | -0.061 |
| AT2G19590 | ACO1 | JA | JA response | -0.014 | -0.067 | 0.122 | -0.061 |
| AT1G05010 | EFE | ET | ET synthesis | 0.983 | -0.287 | 0.133 | -0.898 |
| AT5G03730 | CTR1 | ET | ET signaling | 0.617 | -0.295 | 0.037 | -0.450 |
| AT1G05180 | AXR1 | JA | JA signaling | 0.299 | -0.071 | -0.091 | -0.263 |
| AT2G46370 | JAR1 | JA | aa conjugation of JA | -0.139 | 0.339 | -0.246 | -0.093 |
| AT2G46370 | JAR1 | JA | aa conjugation of JA | 0.335 | 0.027 | -0.431 | -0.106 |
| AT4G15440 | HPL1 | JA | JA response | 0.376 | -0.052 | -0.332 | -0.221 |
| AT4G15440 | HPL1 | JA | JA synthesis | 0.376 | -0.052 | -0.332 | -0.221 |
| AT3G61510 | ACS1 | ET | ET synthesis | -0.070 | -0.052 | -0.057 | -0.121 |
| AT5G65800 | ACS5 | ET | ET synthesis | -0.059 | -0.098 | -0.098 | -0.098 |
| AT3G04580 | EIN4 | ET | ET signaling | 0.218 | -0.233 | -0.066 | -0.358 |
| AT2G43790 | MPK6 | ET | ET signaling | 0.199 | -0.109 | -0.255 | -0.297 |
| AT1G77330 | AT1G77330 | ET | ET synthesis | -0.183 | -0.034 | -0.173 | -0.111 |
| AT4G16760 | ACX1 | JA | JA synthesis | -0.226 | 0.292 | -0.615 | -0.023 |
| AT1G21250 | WAK1 | SA | SA response | -0.003 | 0.116 | 0.148 | -0.853 |
| AT2G46370 | JAR1 | JA | aa conjugation of JA | 0.475 | 0.323 | -0.649 | -0.744 |
| AT1G55180 | PLDEPSILON | SA | link to lipid signaling | -0.188 | -0.214 | -0.131 | -0.343 |
| AT5G20570 | RBX1 | JA | JA signaling | -0.080 | -0.256 | 0.016 | -0.600 |
| AT1G17750 | PEPR2 | JA | JA synthesis | 0.035 | -0.136 | -0.198 | -0.624 |
| AT1G78340 | GSTU22 | JA | oxylipin response | 0.149 | -0.355 | -0.474 | -0.327 |
| AT1G52400 | BGLU18 | JA | JA response | -0.609 | 0.585 | -0.213 | -1.048 |
| AT1G20510 | OPCL1 | JA | JA synthesis | -0.196 | -0.365 | -0.234 | -0.536 |
| AT4G01850 | SAM-2 | ET | ET synthesis | -0.134 | -0.345 | -0.553 | -0.337 |
| AT4G16690 | MES16 | SA | SA synthesis | -0.976 | 0.253 | -0.854 | 0.172 |
| AT3G20770 | EIN3 | ET | ET signaling | -0.260 | -0.520 | -0.382 | -0.440 |
| AT1G72520 | AT1G72520 | JA | JA synthesis | 0.282 | -0.266 | -0.754 | -0.928 |
| AT4G14040 | SBP2 | JA | JA response | -1.897 | -0.390 | -0.143 | 0.742 |
| AT2G32800 | AP4.3A | SA | SA response | -0.185 | -0.460 | -0.521 | -0.596 |
| AT1G19670 | CLH1 | JA | JA response | -1.226 | -0.136 | 0.139 | -0.562 |
| AT1G74100 | SOT16 | JA | JA response | -0.075 | -0.434 | -0.636 | -0.719 |
| AT3G11820 | SYP121 | JA | JA signaling | 0.306 | -0.422 | -0.543 | -1.206 |
| AT3G11820 | SYP121 | SA | SA signaling | 0.306 | -0.422 | -0.543 | -1.206 |
| AT1G73080 | PEPR1 | JA | JA synthesis | 1.051 | -0.146 | -1.314 | -1.493 |
| AT3G44610 | AT3G44610 | JA | JA response | -0.800 | -0.572 | -0.667 | 0.119 |
| AT2G06050 | OPR3 | JA | JA response | -0.341 | -1.317 | -0.219 | -0.339 |
| AT2G06050 | OPR3 | JA | JA synthesis | -0.341 | -1.317 | -0.219 | -0.339 |
| AT3G45640 | MPK3 | ET | ET signaling | -0.381 | -0.453 | -0.641 | -0.857 |
| AT4G34710 | ADC2 | JA | JA response | -0.112 | -0.751 | -1.223 | -0.427 |
| AT4G11830 | PLDGAMMA2 | SA | link to lipid signaling | -0.634 | -0.966 | -0.537 | -0.491 |
| AT5G67300 | MYBR1 | JA | JA signaling | -0.972 | -0.176 | -1.073 | -0.426 |
| AT3G53260 | PAL2 | SA | SA synthesis | -0.868 | -0.150 | -1.429 | -0.365 |
| AT1G64660 | MGL | JA | oxylipin response | 0.050 | -0.182 | -1.234 | -1.466 |
| AT1G51760 | IAR3 | JA | JA response | -0.043 | -0.556 | -0.996 | -1.246 |
| AT4G09900 | MES12 | SA | SA synthesis | -1.882 | -0.400 | -0.567 | -0.006 |
| AT3G25770 | AOC2 | JA | JA response | -0.514 | -1.978 | 0.705 | -1.162 |
| AT3G25770 | AOC2 | JA | JA synthesis | -0.514 | -1.978 | 0.705 | -1.162 |
| AT4G17500 | ERF-1 | JA | JA signaling | 0.134 | -1.012 | -0.604 | -1.506 |
| AT4G17500 | ERF-1 | SA | SA response | 0.134 | -1.012 | -0.604 | -1.506 |
| AT2G25490 | EBF1 | ET | ET signaling | -0.886 | -0.929 | -0.794 | -0.572 |
| AT2G22810 | ACS4 | ET | ET synthesis | -0.940 | -0.857 | -0.700 | -0.794 |
| AT4G34710 | ADC2 | JA | JA response | -0.268 | -0.427 | -1.500 | -1.200 |
| AT3G29770 | MES11 | SA | SA synthesis | -0.678 | -0.517 | -1.347 | -0.951 |
| AT3G25780 | AOC3 | JA | JA synthesis | 0.412 | -0.621 | -1.260 | -2.150 |
| AT4G11830 | PLDGAMMA2 | SA | link to lipid signaling | -1.255 | -1.249 | -0.430 | -0.875 |
| AT3G16640 | TCTP | JA | JA response | -1.232 | -0.969 | -0.986 | -0.712 |
| AT5G42650 | AOS | JA | JA response | -1.060 | -1.011 | -0.810 | -1.099 |
| AT5G42650 | AOS | JA | JA synthesis | -1.060 | -1.011 | -0.810 | -1.099 |
| AT5G04230 | PAL3 | SA | SA synthesis | -1.513 | -0.675 | -1.172 | -0.628 |
| AT1G17420 | LOX3 | JA | JA response | -0.215 | -0.428 | -1.712 | -1.671 |
| AT1G17420 | LOX3 | JA | JA synthesis | -0.215 | -0.428 | -1.712 | -1.671 |
| AT3G10340 | PAL4 | SA | SA synthesis | -0.568 | -0.281 | -1.613 | -1.581 |
| AT3G28740 | CYP81D1 | JA | oxylipin response | -1.336 | -1.221 | -0.303 | -1.197 |
| AT2G23590 | MES8 | SA | SA synthesis | -1.525 | -0.944 | -0.615 | -1.000 |
| AT1G20510 | OPCL1 | JA | JA synthesis | -1.020 | -1.028 | -1.570 | -0.579 |
| AT5G25350 | EBF2 | ET | ET signaling | -0.560 | -1.134 | -1.134 | -1.487 |
| AT4G37770 | ACS8 | ET | ET synthesis | -0.274 | -0.468 | -1.829 | -1.762 |
| AT2G06050 | OPR3 | JA | JA response | -0.977 | -1.183 | -0.820 | -1.491 |
| AT2G06050 | OPR3 | JA | JA synthesis | -0.977 | -1.183 | -0.820 | -1.491 |
| AT5G42720 | AT5G42720 | JA | JA response | -1.152 | -0.333 | -2.030 | -1.340 |
| AT3G11820 | SYP121 | JA | JA signaling | -0.970 | -1.274 | -1.467 | -1.193 |
| AT3G11820 | SYP121 | SA | SA signaling | -0.970 | -1.274 | -1.467 | -1.193 |
| AT3G25760 | AOC1 | JA | JA synthesis | -0.730 | -1.693 | -1.245 | -1.486 |
| AT1G32640 | MYC2 | JA | JA signaling | -1.133 | -1.512 | -1.135 | -1.390 |
| AT5G58310 | MES18 | SA | SA synthesis | -2.112 | -2.241 | -0.505 | -0.424 |
| AT5G24420 | PGL5 | JA | JA response | -1.954 | -1.333 | -1.004 | -1.031 |
| AT3G22400 | LOX5 | JA | JA synthesis | -0.920 | -1.232 | -1.568 | -1.706 |
| AT4G11280 | ACS6 | ET | ET synthesis | -0.368 | -0.349 | -2.228 | -2.495 |
| AT5G04230 | PAL3 | SA | SA synthesis | -2.168 | -1.434 | -1.152 | -0.880 |
| AT4G18440 | AT4G18440 | JA | JA response | -2.977 | -1.582 | -0.946 | -0.995 |
| AT2G06050 | OPR3 | JA | JA response | -1.284 | -1.786 | -1.366 | -2.101 |
| AT2G06050 | OPR3 | JA | JA synthesis | -1.284 | -1.786 | -1.366 | -2.101 |
| AT2G27050 | EIL1 | ET | ET signaling | -1.571 | -1.950 | -1.436 | -1.637 |
| AT3G17390 | MTO3 | ET | ET synthesis | -1.723 | -1.575 | -2.328 | -1.092 |
| AT1G12010 | AT1G12010 | ET | ET synthesis | -1.822 | -1.833 | -1.691 | -1.640 |
| AT2G47730 | GSTF8 | JA | oxylipin response | -1.355 | -1.501 | -2.360 | -1.930 |
| AT1G19640 | JMT | JA | JA synthesis | -1.262 | -0.894 | -2.699 | -2.407 |
| AT1G76680 | OPR1 | JA | JA response | -1.895 | -1.490 | -1.917 | -2.141 |
| AT1G76680 | OPR1 | JA | JA synthesis | -1.895 | -1.490 | -1.917 | -2.141 |
| AT1G76680 | OPR1 | JA | oxylipin response | -1.895 | -1.490 | -1.917 | -2.141 |
| AT4G08040 | ACS11 | ET | ET synthesis | -1.447 | -2.059 | -2.552 | -2.165 |
| AT1G73500 | MKK9 | ET | ET signaling | -2.041 | -2.623 | -2.061 | -2.598 |
| AT1G76680 | OPR1 | JA | JA response | -1.295 | -2.461 | -2.912 | -2.962 |
| AT1G76680 | OPR1 | JA | JA synthesis | -1.295 | -2.461 | -2.912 | -2.962 |
| AT1G76680 | OPR1 | JA | oxylipin response | -1.295 | -2.461 | -2.912 | -2.962 |
| AT2G23560 | MES7 | SA | SA synthesis | -1.954 | -2.495 | -2.537 | -2.658 |
