# Supplementary material for: The transcriptome analysis of the Arabidopsis thaliana in response to the Vibrio vulnificus by RNA-sequencing
Source: PLoS One. 2019 Dec 16;14(12):e0225976. doi: 10.1371/journal.pone.0225976 (PMC6913959; doi:10.1371/journal.pone.0225976)
Supplement: S7 Table — (DOCX) [file pone.0225976.s009.docx]

| **S7 Table.** Overlapped DEGs between *P. syringae* and *V. vulnificus* 96-11-17M infiltrarion. | | | | | | |  |
| --- | --- | --- | --- | --- | --- | --- | --- |
|  | **24 h** |  |  |  |  | **48 h** |  |
| **RNAseq.tracking_id** | **Gene Symbol** | **FC log ratio(24h/0h)** |  |  | **RNA-seq.tracking_id** | **Gene Symbol** | **FC.logratio(48h/0h)** |
| PR1 | PR1 | 7.54 |  |  | PR1 | PR1 | 9.80 |
| FRK1 | FRK1 | 6.57 |  |  | CYP71A12 | CYP71A12 | 7.32 |
| NRT2.6 | NRT2.6 | 6.22 |  |  | CCR2 | CCR2 | 6.99 |
| SAG13 | SAG13 | 6.04 |  |  | ELF4 | ELF4 | 6.78 |
| PROPEP3 | PROPEP3 | 5.75 |  |  | FRK1 | FRK1 | 6.37 |
| YLS9 | YLS9 | 5.30 |  |  | NRT2.6 | NRT2.6 | 6.31 |
| Rap2.6L | Rap2.6L | 5.23 |  |  | YLS9 | YLS9 | 5.92 |
| WRKY60 | WRKY60 | 5.18 |  |  | ELI3-2 | ELI3-2 | 5.76 |
| GPT2 | GPT2 | 5.09 |  |  | GPT2 | GPT2 | 5.71 |
| ACS2 | ACS2 | 5.01 |  |  | GSTU10 | GSTU10 | 5.67 |
| AAT1 | AAT1 | 4.93 |  |  | AOX1D | AOX1D | 5.63 |
| AOX1D | AOX1D | 4.92 |  |  | SAG13 | SAG13 | 5.55 |
| DIN2 | DIN2 | 4.80 |  |  | DIN2 | DIN2 | 5.19 |
| WRKY75 | WRKY75 | 4.78 |  |  | DOX1 | DOX1 | 5.16 |
| CRK13 | CRK13 | 4.76 |  |  | AGP2 | AGP2 | 5.01 |
| WR3 | WR3 | 4.72 |  |  | PDF1.2 | PDF1.2 | 4.91 |
| GSTU10 | GSTU10 | 4.71 |  |  | CAX3 | CAX3 | 4.89 |
| CAX3 | CAX3 | 4.68 |  |  | PROPEP3 | PROPEP3 | 4.81 |
| PR4 | PR4 | 4.63 |  |  | WRKY75 | WRKY75 | 4.77 |
| KOR2 | KOR2 | 4.53 |  |  | WR3 | WR3 | 4.76 |
| PTR3 | PTR3 | 4.34 |  |  | PTR3 | PTR3 | 4.60 |
| SRG1 | SRG1 | 4.17 |  |  | PAD3 | PAD3 | 4.52 |
| MPL1 | MPL1 | 4.13 |  |  | CYP76C2 | CYP76C2 | 4.50 |
| SBT3.5 | SBT3.5 | 4.12 |  |  | WRKY60 | WRKY60 | 4.43 |
| PBS3 | PBS3 | 4.07 |  |  | PR4 | PR4 | 4.31 |
| AIG1 | AIG1 | 3.79 |  |  | MLO12 | MLO12 | 4.29 |
| PXMT1 | PXMT1 | 3.60 |  |  | BGLU44 | BGLU44 | 4.24 |
| GH3.3 | GH3.3 | 3.58 |  |  | PHS2 | PHS2 | 4.18 |
| PAD3 | PAD3 | 3.56 |  |  | WRKY61 | WRKY61 | 4.10 |
| CSLG2 | CSLG2 | 3.44 |  |  | AAT1 | AAT1 | 4.03 |
| CYP710A1 | CYP710A1 | 3.42 |  |  | COR15B | COR15B | 4.03 |
| CYP81G1 | CYP81G1 | 3.41 |  |  | ALIS5 | ALIS5 | 3.94 |
| ALIS5 | ALIS5 | 3.40 |  |  | SRG1 | SRG1 | 3.94 |
| PAP17 | PAP17 | 3.39 |  |  | Rap2.6L | Rap2.6L | 3.94 |
| GSR 1 | GSR 1 | 3.36 |  |  | DREB1A | DREB1A | 3.86 |
| ELF4 | ELF4 | 3.35 |  |  | SBT3.5 | SBT3.5 | 3.84 |
| PDF1.2 | PDF1.2 | 3.34 |  |  | WRKY45 | WRKY45 | 3.83 |
| SBT3.3 | SBT3.3 | 3.33 |  |  | LZF1 | LZF1 | 3.83 |
| CYP71A13 | CYP71A13 | 3.17 |  |  | CYP71A13 | CYP71A13 | 3.82 |
| WRKY45 | WRKY45 | 3.14 |  |  | WAK3 | WAK3 | 3.78 |
| NIMIN1 | NIMIN1 | 3.13 |  |  | GI | GI | 3.76 |
| WRKY6 | WRKY6 | 3.13 |  |  | ACS2 | ACS2 | 3.72 |
| HSP17.6II | HSP17.6II | 3.09 |  |  | AT14A | AT14A | 3.70 |
| EDL3 | EDL3 | 3.08 |  |  | AAC2 | AAC2 | 3.63 |
| WRKY38 | WRKY38 | 3.05 |  |  | DPE1 | DPE1 | 3.61 |
| tic20-IV | tic20-IV | 2.99 |  |  | CSLG2 | CSLG2 | 3.55 |
| MYB2 | MYB2 | 2.97 |  |  | DXPS1 | DXPS1 | 3.54 |
| SQE6 | SQE6 | 2.95 |  |  | ACA8 | ACA8 | 3.53 |
| WRKY9 | WRKY9 | 2.93 |  |  | SEX1 | SEX1 | 3.45 |
| ARPN | ARPN | 2.90 |  |  | PDR12 | PDR12 | 3.44 |
| CYP81D8 | CYP81D8 | 2.88 |  |  | PAP17 | PAP17 | 3.41 |
| UGT76E12 | UGT76E12 | 2.83 |  |  | ALDH2B7 | ALDH2B7 | 3.37 |
| WAK3 | WAK3 | 2.82 |  |  | AMY1 | AMY1 | 3.26 |
| PDR12 | PDR12 | 2.80 |  |  | WRKY38 | WRKY38 | 3.26 |
| GLP5 | GLP5 | 2.79 |  |  | MES9 | MES9 | 3.18 |
| LHT7 | LHT7 | 2.79 |  |  | HSP17.4 | HSP17.4 | 3.13 |
| ST2A | ST2A | 2.67 |  |  | WRKY31 | WRKY31 | 3.07 |
| WRKY30 | WRKY30 | 2.62 |  |  | BGAL10 | BGAL10 | 3.07 |
| WRKY28 | WRKY28 | 2.62 |  |  | PDR9 | PDR9 | 3.05 |
| ATBFRUCT1 | ATBFRUCT1 | 2.60 |  |  | MPL1 | MPL1 | 3.02 |
| MOT1 | MOT1 | 2.53 |  |  | GLR2.5 | GLR2.5 | 3.01 |
| BGLU46 | BGLU46 | 2.52 |  |  | AAP3 | AAP3 | 3.01 |
| RAP2.6 | RAP2.6 | 2.52 |  |  | CYP710A1 | CYP710A1 | 2.98 |
| AGP5 | AGP5 | 2.48 |  |  | ADH1 | ADH1 | 2.97 |
| XTR8 | XTR8 | 2.47 |  |  | PORB | PORB | 2.88 |
| BSMT1 | BSMT1 | 2.47 |  |  | WRKY6 | WRKY6 | 2.85 |
| LECRKA4.1 | LECRKA4.1 | 2.46 |  |  | CYP81G1 | CYP81G1 | 2.85 |
| CYP71B23 | CYP71B23 | 2.46 |  |  | PBS3 | PBS3 | 2.79 |
| GLR2.5 | GLR2.5 | 2.42 |  |  | tic20-IV | tic20-IV | 2.77 |
| MYB15 | MYB15 | 2.42 |  |  | SBT3.3 | SBT3.3 | 2.76 |
| DPL1 | DPL1 | 2.38 |  |  | AMY3 | AMY3 | 2.74 |
| PROPEP2 | PROPEP2 | 2.37 |  |  | XTR8 | XTR8 | 2.72 |
| ALD1 | ALD1 | 2.37 |  |  | ACD32.1 | ACD32.1 | 2.68 |
| FMO1 | FMO1 | 2.34 |  |  | CYP81D8 | CYP81D8 | 2.63 |
| SD1-29 | SD1-29 | 2.31 |  |  | AIG1 | AIG1 | 2.60 |
| BIP2 | BIP2 | 2.30 |  |  | MAPKKK18 | MAPKKK18 | 2.58 |
| 4CL5 | 4CL5 | 2.30 |  |  | DPE2 | DPE2 | 2.57 |
| UGT73C7 | UGT73C7 | 2.29 |  |  | BG3 | BG3 | 2.54 |
| BGLU45 | BGLU45 | 2.27 |  |  | CYP76C1 | CYP76C1 | 2.44 |
| FIB2 | FIB2 | 2.21 |  |  | GH3.3 | GH3.3 | 2.42 |
| PMZ | PMZ | 2.21 |  |  | DFL1 | DFL1 | 2.40 |
| YLS2 | YLS2 | 2.20 |  |  | MEE18 | MEE18 | 2.38 |
| PLA2A | PLA2A | 2.18 |  |  | AGP5 | AGP5 | 2.38 |
| LTP4 | LTP4 | 2.17 |  |  | FMO1 | FMO1 | 2.31 |
| MC8 | MC8 | 2.17 |  |  | ATBFRUCT1 | ATBFRUCT1 | 2.27 |
| BR6OX2 | BR6OX2 | 2.15 |  |  | YLS5 | YLS5 | 2.24 |
| AMY1 | AMY1 | 2.12 |  |  | MYB2 | MYB2 | 2.22 |
| LTI65 | LTI65 | 2.10 |  |  | BGLU46 | BGLU46 | 2.21 |
| NDB2 | NDB2 | 2.10 |  |  | DFR | DFR | 2.19 |
| BAP2 | BAP2 | 2.08 |  |  | NAS3 | NAS3 | 2.18 |
| FPF1 | FPF1 | 2.04 |  |  | HSF4 | HSF4 | 2.11 |
| AATP1 | AATP1 | 2.03 |  |  | LTI78 | LTI78 | 2.09 |
| HSF4 | HSF4 | 1.97 |  |  | LHT7 | LHT7 | 2.07 |
| YLS5 | YLS5 | 1.97 |  |  | UGT73D1 | UGT73D1 | 2.05 |
| HSP70 | HSP70 | 1.94 |  |  | WRKY28 | WRKY28 | 2.04 |
| HDT4 | HDT4 | 1.92 |  |  | GER3 | GER3 | 2.03 |
| TPS04 | TPS04 | 1.87 |  |  | FRA8 | FRA8 | 2.02 |
| PI | PI | 1.87 |  |  | PLDP2 | PLDP2 | 2.01 |
| GI | GI | 1.87 |  |  | APS4 | APS4 | 2.00 |
| ATERDJ3A | ATERDJ3A | 1.77 |  |  | DPL1 | DPL1 | 1.97 |
| G6PD6 | G6PD6 | 1.77 |  |  | DIN11 | DIN11 | 1.97 |
| CYP81D1 | CYP81D1 | 1.77 |  |  | YLS2 | YLS2 | 1.94 |
| CNX1 | CNX1 | 1.76 |  |  | RNS1 | RNS1 | 1.94 |
| WRKY55 | WRKY55 | 1.75 |  |  | AIG2 | AIG2 | 1.93 |
| MMP | MMP | 1.73 |  |  | SHA1 | SHA1 | 1.91 |
| BRL3 | BRL3 | 1.72 |  |  | scpl14 | scpl14 | 1.89 |
| UGT73D1 | UGT73D1 | 1.71 |  |  | GID1B | GID1B | 1.87 |
| FMO GS-OX4 | FMO GS-OX4 | 1.68 |  |  | APL2 | APL2 | 1.85 |
| RHF1A | RHF1A | 1.67 |  |  | CYP71B23 | CYP71B23 | 1.84 |
| SQP1 | SQP1 | 1.65 |  |  | UF3GT | UF3GT | 1.84 |
| EDS16 | EDS16 | 1.64 |  |  | COR413-PM1 | COR413-PM1 | 1.84 |
| LACS7 | LACS7 | 1.63 |  |  | HSP17.6II | HSP17.6II | 1.80 |
| OPT1 | OPT1 | 1.61 |  |  | ILR1 | ILR1 | 1.80 |
| TAT3 | TAT3 | 1.61 |  |  | CYP71B36 | CYP71B36 | 1.79 |
| ZIP11 | ZIP11 | 1.57 |  |  | TRANS11 | TRANS11 | 1.79 |
| UGE5 | UGE5 | 1.57 |  |  | TSA1 | TSA1 | 1.78 |
| MYB122 | MYB122 | 1.56 |  |  | CBP60G | CBP60G | 1.77 |
| TGA1 | TGA1 | 1.56 |  |  | NAC1 | NAC1 | 1.76 |
| WAKL7 | WAKL7 | 1.55 |  |  | BGLU9 | BGLU9 | 1.76 |
| CYP89A9 | CYP89A9 | 1.54 |  |  | DBE1 | DBE1 | 1.73 |
| WRKY27 | WRKY27 | 1.54 |  |  | VSP1 | VSP1 | 1.72 |
| GAPC1 | GAPC1 | 1.53 |  |  | SD1-29 | SD1-29 | 1.71 |
| ABCB4 | ABCB4 | 1.52 |  |  | PLA2-ALPHA | PLA2-ALPHA | 1.70 |
| TET3 | TET3 | 1.51 |  |  | TET3 | TET3 | 1.67 |
| CAX7 | CAX7 | 1.50 |  |  | BAM5 | BAM5 | 1.67 |
| CBP60G | CBP60G | 1.50 |  |  | AATP1 | AATP1 | 1.65 |
| RHD2 | RHD2 | 1.49 |  |  | NSP5 | NSP5 | 1.64 |
| UNE5 | UNE5 | 1.49 |  |  | CAM8 | CAM8 | 1.63 |
| UTR3 | UTR3 | 1.49 |  |  | LTI65 | LTI65 | 1.61 |
| TSA1 | TSA1 | 1.45 |  |  | COPT2 | COPT2 | 1.55 |
| CRCK1 | CRCK1 | 1.45 |  |  | CYP89A9 | CYP89A9 | 1.51 |
| AIG2 | AIG2 | 1.43 |  |  | LECRKA4.1 | LECRKA4.1 | 1.49 |
| WRKY67 | WRKY67 | 1.43 |  |  | 4CL5 | 4CL5 | 1.48 |
| BGLU11 | BGLU11 | 1.42 |  |  | CRCK1 | CRCK1 | 1.48 |
| MBF1C | MBF1C | 1.41 |  |  | ELF4-L3 | ELF4-L3 | 1.47 |
| MYB85 | MYB85 | 1.40 |  |  | SCPL7 | SCPL7 | 1.46 |
| FSD3 | FSD3 | 1.40 |  |  | CYP706A7 | CYP706A7 | 1.44 |
| GID1B | GID1B | 1.37 |  |  | ABCB4 | ABCB4 | 1.42 |
| MYB37 | MYB37 | 1.37 |  |  | PRF4 | PRF4 | 1.42 |
| WRKY46 | WRKY46 | 1.35 |  |  | LACS7 | LACS7 | 1.41 |
| WNK4 | WNK4 | 1.34 |  |  | NAM | NAM | 1.40 |
| BGLU44 | BGLU44 | 1.33 |  |  | EDS16 | EDS16 | 1.40 |
| SAG21 | SAG21 | 1.33 |  |  | scpl16 | scpl16 | 1.40 |
| WRKY11 | WRKY11 | 1.32 |  |  | SGS3 | SGS3 | 1.39 |
| LTP3 | LTP3 | 1.32 |  |  | PLC4 | PLC4 | 1.37 |
| ERD1 | ERD1 | 1.31 |  |  | ERD3 | ERD3 | 1.36 |
| UNE18 | UNE18 | 1.30 |  |  | sks15 | sks15 | 1.33 |
| BETA-VPE | BETA-VPE | 1.30 |  |  | GAPC1 | GAPC1 | 1.32 |
| BGLU9 | BGLU9 | 1.30 |  |  | DELTA-OAT | DELTA-OAT | 1.32 |
| LTI30 | LTI30 | 1.29 |  |  | CYP81D1 | CYP81D1 | 1.32 |
| RACK1C_AT | RACK1C_AT | 1.28 |  |  | BAC2 | BAC2 | 1.31 |
| CBF4 | CBF4 | 1.26 |  |  | AIR3 | AIR3 | 1.31 |
| BAM5 | BAM5 | 1.25 |  |  | LTI30 | LTI30 | 1.30 |
| DREB1A | DREB1A | 1.25 |  |  | ABI1 | ABI1 | 1.30 |
| SFR6 | SFR6 | 1.23 |  |  | WRKY26 | WRKY26 | 1.28 |
| DBE1 | DBE1 | 1.19 |  |  | ABI5 | ABI5 | 1.27 |
| SCL3 | SCL3 | 1.19 |  |  | MLO3 | MLO3 | 1.26 |
| SGP1 | SGP1 | 1.18 |  |  | TGA1 | TGA1 | 1.26 |
| ATERDJ3B | ATERDJ3B | 1.16 |  |  | MYB15 | MYB15 | 1.25 |
| CYP706A5 | CYP706A5 | 1.15 |  |  | ATCSLA15 | ATCSLA15 | 1.22 |
| UGT71B6 | UGT71B6 | 1.14 |  |  | UTR1 | UTR1 | 1.21 |
| WRKY47 | WRKY47 | 1.13 |  |  | SRG3 | SRG3 | 1.20 |
| APG8A | APG8A | 1.13 |  |  | RFC3 | RFC3 | 1.20 |
| CHX18 | CHX18 | 1.13 |  |  | BGLU11 | BGLU11 | 1.18 |
| SDF2 | SDF2 | 1.12 |  |  | CHAT | CHAT | 1.18 |
| MYB14 | MYB14 | 1.12 |  |  | APG8A | APG8A | 1.17 |
| AAC3 | AAC3 | 1.12 |  |  | TMT2 | TMT2 | 1.16 |
| SUS3 | SUS3 | 1.11 |  |  | NF-YA10 | NF-YA10 | 1.16 |
| EDA39 | EDA39 | 1.09 |  |  | NDB2 | NDB2 | 1.16 |
| SUMO3 | SUMO3 | 1.09 |  |  | NRAMP1 | NRAMP1 | 1.16 |
| EMB2729 | EMB2729 | 1.08 |  |  | PGM | PGM | 1.16 |
| CAM8 | CAM8 | 1.07 |  |  | CCB3 | CCB3 | 1.15 |
| NF-YC7 | NF-YC7 | 1.07 |  |  | BGL2 | BGL2 | 1.15 |
| EDS5 | EDS5 | 1.07 |  |  | EXL4 | EXL4 | 1.15 |
| NDA2 | NDA2 | 1.05 |  |  | OPT1 | OPT1 | 1.11 |
| PHS2 | PHS2 | 1.05 |  |  | TPS04 | TPS04 | 1.09 |
| CCR2 | CCR2 | 1.02 |  |  | COR15A | COR15A | 1.09 |
| ATLP-1 | ATLP-1 | -1.00 |  |  | PLDBETA2 | PLDBETA2 | 1.08 |
| MLP423 | MLP423 | -1.01 |  |  | TRP1 | TRP1 | 1.08 |
| DEGP2 | DEGP2 | -1.01 |  |  | CYP707A4 | CYP707A4 | 1.07 |
| SCPL13 | SCPL13 | -1.02 |  |  | UGT73B5 | UGT73B5 | 1.06 |
| ICS2 | ICS2 | -1.03 |  |  | BGLU8 | BGLU8 | 1.05 |
| SQE3 | SQE3 | -1.03 |  |  | MES17 | MES17 | 1.05 |
| NDF4 | NDF4 | -1.03 |  |  | QRT2 | QRT2 | 1.05 |
| BGAL2 | BGAL2 | -1.04 |  |  | UGE5 | UGE5 | 1.05 |
| 4CL3 | 4CL3 | -1.04 |  |  | WNK4 | WNK4 | 1.04 |
| FKBP13 | FKBP13 | -1.04 |  |  | PR5 | PR5 | 1.03 |
| NDF5 | NDF5 | -1.04 |  |  | SS2 | SS2 | 1.02 |
| ATL4 | ATL4 | -1.04 |  |  | MAP18 | MAP18 | 1.01 |
| NUDT7 | NUDT7 | -1.06 |  |  | LHCB6 | LHCB6 | -1.00 |
| PCB2 | PCB2 | -1.07 |  |  | AGL87 | AGL87 | -1.02 |
| HAE | HAE | -1.08 |  |  | 2A6 | 2A6 | -1.02 |
| AGL87 | AGL87 | -1.09 |  |  | ACP1 | ACP1 | -1.02 |
| FLS2 | FLS2 | -1.09 |  |  | GA2OX6 | GA2OX6 | -1.03 |
| CPK28 | CPK28 | -1.09 |  |  | CTF2A | CTF2A | -1.04 |
| RPL9 | RPL9 | -1.11 |  |  | ABCA4 | ABCA4 | -1.05 |
| PUB18 | PUB18 | -1.13 |  |  | UGT73C6 | UGT73C6 | -1.06 |
| AGP9 | AGP9 | -1.13 |  |  | CYP81F2 | CYP81F2 | -1.08 |
| EBF2 | EBF2 | -1.13 |  |  | CRF6 | CRF6 | -1.09 |
| 2A6 | 2A6 | -1.14 |  |  | HT1 | HT1 | -1.09 |
| FUT13 | FUT13 | -1.14 |  |  | WSD1 | WSD1 | -1.10 |
| LHCB6 | LHCB6 | -1.14 |  |  | PIF4 | PIF4 | -1.10 |
| GHS1 | GHS1 | -1.16 |  |  | OVA4 | OVA4 | -1.11 |
| SCRM2 | SCRM2 | -1.16 |  |  | EBF2 | EBF2 | -1.13 |
| AGP14 | AGP14 | -1.19 |  |  | EOL2 | EOL2 | -1.14 |
| OVA4 | OVA4 | -1.20 |  |  | STO | STO | -1.15 |
| FLA8 | FLA8 | -1.21 |  |  | IAGLU | IAGLU | -1.15 |
| LOX5 | LOX5 | -1.23 |  |  | PDS1 | PDS1 | -1.16 |
| CLE17 | CLE17 | -1.24 |  |  | ATLP-1 | ATLP-1 | -1.16 |
| ETC2 | ETC2 | -1.25 |  |  | OBP3 | OBP3 | -1.18 |
| SP1L4 | SP1L4 | -1.26 |  |  | PROPEP4 | PROPEP4 | -1.18 |
| BGAL5 | BGAL5 | -1.27 |  |  | iqd21 | iqd21 | -1.19 |
| LHCB3 | LHCB3 | -1.28 |  |  | GASA4 | GASA4 | -1.20 |
| ARR4 | ARR4 | -1.32 |  |  | SAUR15 | SAUR15 | -1.21 |
| TUB1 | TUB1 | -1.32 |  |  | MEE23 | MEE23 | -1.24 |
| ATPRX Q | ATPRX Q | -1.33 |  |  | LHCB3 | LHCB3 | -1.25 |
| RALFL34 | RALFL34 | -1.33 |  |  | BUD2 | BUD2 | -1.29 |
| COR47 | COR47 | -1.35 |  |  | LHCA4 | LHCA4 | -1.31 |
| CAD9 | CAD9 | -1.37 |  |  | TINY2 | TINY2 | -1.35 |
| F3H | F3H | -1.37 |  |  | PUB18 | PUB18 | -1.37 |
| APX4 | APX4 | -1.37 |  |  | FUC95A | FUC95A | -1.38 |
| ACA1 | ACA1 | -1.38 |  |  | SKS6 | SKS6 | -1.40 |
| PDX1.1 | PDX1.1 | -1.39 |  |  | WRKY18 | WRKY18 | -1.41 |
| DAR3 | DAR3 | -1.39 |  |  | BGAL8 | BGAL8 | -1.43 |
| AGP12 | AGP12 | -1.41 |  |  | CYP71B13 | CYP71B13 | -1.44 |
| ARR9 | ARR9 | -1.42 |  |  | CER1 | CER1 | -1.47 |
| TAR2 | TAR2 | -1.42 |  |  | FLA8 | FLA8 | -1.49 |
| ELIP2 | ELIP2 | -1.43 |  |  | FLA15 | FLA15 | -1.49 |
| ARL | ARL | -1.44 |  |  | AGP4 | AGP4 | -1.50 |
| FLA13 | FLA13 | -1.45 |  |  | ADC2 | ADC2 | -1.50 |
| HTH | HTH | -1.46 |  |  | LPD1 | LPD1 | -1.50 |
| tny | tny | -1.46 |  |  | UGT74F2 | UGT74F2 | -1.51 |
| LACS2 | LACS2 | -1.46 |  |  | FLS2 | FLS2 | -1.51 |
| DDF1 | DDF1 | -1.47 |  |  | IRX1 | IRX1 | -1.53 |
| DRT100 | DRT100 | -1.48 |  |  | OFP16 | OFP16 | -1.55 |
| MIPS1 | MIPS1 | -1.50 |  |  | BT2 | BT2 | -1.57 |
| NIK1 | NIK1 | -1.50 |  |  | LOX5 | LOX5 | -1.57 |
| TT4 | TT4 | -1.51 |  |  | GUN5 | GUN5 | -1.61 |
| ERD6 | ERD6 | -1.51 |  |  | GASA1 | GASA1 | -1.62 |
| RALFL24 | RALFL24 | -1.52 |  |  | ABCB19 | ABCB19 | -1.63 |
| IQD11 | IQD11 | -1.53 |  |  | JAZ10 | JAZ10 | -1.64 |
| MEE3 | MEE3 | -1.54 |  |  | CER4 | CER4 | -1.67 |
| GASA4 | GASA4 | -1.55 |  |  | LHCB4.2 | LHCB4.2 | -1.67 |
| iqd21 | iqd21 | -1.55 |  |  | MLP28 | MLP28 | -1.67 |
| LHCA4 | LHCA4 | -1.57 |  |  | TIFY10B | TIFY10B | -1.68 |
| UGT73B1 | UGT73B1 | -1.58 |  |  | HTH | HTH | -1.69 |
| LHCA6 | LHCA6 | -1.58 |  |  | NIK1 | NIK1 | -1.69 |
| ACP4 | ACP4 | -1.59 |  |  | CIL | CIL | -1.71 |
| AGP21 | AGP21 | -1.60 |  |  | PDLP2 | PDLP2 | -1.72 |
| COL2 | COL2 | -1.61 |  |  | LHCB2.1 | LHCB2.1 | -1.72 |
| BDG1 | BDG1 | -1.61 |  |  | RCI2B | RCI2B | -1.74 |
| F8H | F8H | -1.62 |  |  | IQD11 | IQD11 | -1.76 |
| ERF2 | ERF2 | -1.63 |  |  | TUB7 | TUB7 | -1.79 |
| GER3 | GER3 | -1.68 |  |  | PDLP3 | PDLP3 | -1.79 |
| RGL3 | RGL3 | -1.69 |  |  | RALFL34 | RALFL34 | -1.84 |
| COL5 | COL5 | -1.74 |  |  | APT5 | APT5 | -1.85 |
| BEN1 | BEN1 | -1.74 |  |  | APK1A | APK1A | -1.85 |
| CML38 | CML38 | -1.77 |  |  | ATAUX2-11 | ATAUX2-11 | -1.85 |
| BGLU6 | BGLU6 | -1.77 |  |  | PLP9 | PLP9 | -1.86 |
| LGT8 | LGT8 | -1.77 |  |  | MEE3 | MEE3 | -1.88 |
| PDLP3 | PDLP3 | -1.78 |  |  | SEN1 | SEN1 | -1.92 |
| TMK1 | TMK1 | -1.81 |  |  | LBD38 | LBD38 | -1.92 |
| LBD38 | LBD38 | -1.85 |  |  | GGPS6 | GGPS6 | -1.95 |
| PGP21 | PGP21 | -1.87 |  |  | SAUR68 | SAUR68 | -1.96 |
| PIF4 | PIF4 | -1.88 |  |  | DRT100 | DRT100 | -1.98 |
| LHCB4.2 | LHCB4.2 | -1.89 |  |  | LACS2 | LACS2 | -1.99 |
| BMY3 | BMY3 | -1.90 |  |  | XCP1 | XCP1 | -2.01 |
| PRE1 | PRE1 | -1.90 |  |  | ELIP2 | ELIP2 | -2.02 |
| GLYR2 | GLYR2 | -1.93 |  |  | ARL | ARL | -2.06 |
| AMP1 | AMP1 | -1.94 |  |  | PRE1 | PRE1 | -2.07 |
| SQS2 | SQS2 | -1.97 |  |  | ZW9 | ZW9 | -2.09 |
| OFP16 | OFP16 | -1.98 |  |  | RAP2.10 | RAP2.10 | -2.12 |
| ACR4 | ACR4 | -1.98 |  |  | GPAT8 | GPAT8 | -2.14 |
| CRF2 | CRF2 | -2.00 |  |  | HFR1 | HFR1 | -2.14 |
| FLA9 | FLA9 | -2.02 |  |  | ABA1 | ABA1 | -2.20 |
| AGP4 | AGP4 | -2.04 |  |  | PDX1.1 | PDX1.1 | -2.20 |
| CYP86A8 | CYP86A8 | -2.06 |  |  | CRF2 | CRF2 | -2.22 |
| MYB77 | MYB77 | -2.07 |  |  | BEN1 | BEN1 | -2.22 |
| IAA14 | IAA14 | -2.07 |  |  | ACS6 | ACS6 | -2.23 |
| ABCB19 | ABCB19 | -2.08 |  |  | ACR3 | ACR3 | -2.23 |
| ELP | ELP | -2.09 |  |  | PGP21 | PGP21 | -2.24 |
| ERD5 | ERD5 | -2.09 |  |  | BGLU6 | BGLU6 | -2.27 |
| HHP1 | HHP1 | -2.11 |  |  | ACA1 | ACA1 | -2.33 |
| LAC8 | LAC8 | -2.13 |  |  | CYP86A8 | CYP86A8 | -2.34 |
| PHO1 | PHO1 | -2.19 |  |  | DDF1 | DDF1 | -2.39 |
| MYB73 | MYB73 | -2.21 |  |  | NAP | NAP | -2.43 |
| ARR6 | ARR6 | -2.24 |  |  | EXPA5 | EXPA5 | -2.45 |
| SAUR68 | SAUR68 | -2.24 |  |  | THI2.2 | THI2.2 | -2.52 |
| MES18 | MES18 | -2.24 |  |  | SQS2 | SQS2 | -2.55 |
| EXL5 | EXL5 | -2.25 |  |  | SP1L4 | SP1L4 | -2.58 |
| PKS2 | PKS2 | -2.36 |  |  | UGT74D1 | UGT74D1 | -2.69 |
| KCS8 | KCS8 | -2.36 |  |  | TMAC2 | TMAC2 | -2.74 |
| TUB5 | TUB5 | -2.37 |  |  | AGP7 | AGP7 | -2.80 |
| PHI-1 | PHI-1 | -2.37 |  |  | PGP14 | PGP14 | -2.83 |
| JAZ7 | JAZ7 | -2.37 |  |  | ATCSLA09 | ATCSLA09 | -2.89 |
| THI2.2 | THI2.2 | -2.38 |  |  | KCS5 | KCS5 | -2.93 |
| ARR7 | ARR7 | -2.40 |  |  | AGP21 | AGP21 | -2.93 |
| EXPA5 | EXPA5 | -2.45 |  |  | LHCB2.3 | LHCB2.3 | -2.96 |
| GPAT8 | GPAT8 | -2.45 |  |  | PAR1 | PAR1 | -3.03 |
| RRTF1 | RRTF1 | -2.50 |  |  | XTH9 | XTH9 | -3.04 |
| GPAT3 | GPAT3 | -2.65 |  |  | FLA13 | FLA13 | -3.04 |
| CYP86A2 | CYP86A2 | -2.66 |  |  | NAI1 | NAI1 | -3.05 |
| GRH1 | GRH1 | -2.68 |  |  | CAD9 | CAD9 | -3.06 |
| AGP26 | AGP26 | -2.69 |  |  | BZIP34 | BZIP34 | -3.16 |
| KCS1 | KCS1 | -2.70 |  |  | EXL5 | EXL5 | -3.23 |
| XTH9 | XTH9 | -2.71 |  |  | ULT1 | ULT1 | -3.24 |
| NAI1 | NAI1 | -2.82 |  |  | AGP17 | AGP17 | -3.45 |
| SBH2 | SBH2 | -2.82 |  |  | ERD5 | ERD5 | -3.45 |
| AFO | AFO | -2.84 |  |  | COL1 | COL1 | -3.47 |
| LHCB2.3 | LHCB2.3 | -2.85 |  |  | ARR6 | ARR6 | -3.49 |
| SHY2 | SHY2 | -2.89 |  |  | IAA6 | IAA6 | -3.56 |
| XTH33 | XTH33 | -2.98 |  |  | RRTF1 | RRTF1 | -3.67 |
| CYP94C1 | CYP94C1 | -3.07 |  |  | MIPS1 | MIPS1 | -3.88 |
| CYP707A3 | CYP707A3 | -3.12 |  |  | NDA1 | NDA1 | -3.95 |
| BT5 | BT5 | -3.14 |  |  | CYP94C1 | CYP94C1 | -3.96 |
| CER2 | CER2 | -3.24 |  |  | SHY2 | SHY2 | -3.97 |
| IAA6 | IAA6 | -3.37 |  |  | HAT1 | HAT1 | -4.18 |
| DELTA-TIP | DELTA-TIP | -3.41 |  |  | BMY3 | BMY3 | -4.20 |
| BT2 | BT2 | -3.68 |  |  | CER2 | CER2 | -4.46 |
| HAT1 | HAT1 | -4.00 |  |  | DELTA-TIP | DELTA-TIP | -4.57 |
| PAR1 | PAR1 | -4.01 |  |  | COL2 | COL2 | -4.62 |
| EXL3 | EXL3 | -4.14 |  |  | GPAT3 | GPAT3 | -4.82 |
| BZIP61 | BZIP61 | -4.24 |  |  | PHI-1 | PHI-1 | -6.11 |
| BXL2 | BXL2 | -5.08 |  |  |  |  |  |
